# The fine‐scale genetic structure and selection signals of Chinese indigenous pigs

**DOI:** 10.1111/eva.12887

**Published:** 2019-11-22

**Authors:** Min Huang, Bin Yang, Hao Chen, Hui Zhang, Zhongping Wu, Huashui Ai, Jun Ren, Lusheng Huang

**Affiliations:** ^1^ State Key Laboratory of Pig Genetic Improvement and Production Technology Jiangxi Agricultural University Nanchang China; ^2^ Present address: College of Animal Science South China Agricultural University Guangzhou China

**Keywords:** Chinese pig, coat color, genetic diversity, high‐altitude adaptation, phylogenetic relationship, population structure

## Abstract

Genome‐wide SNP profiling has yielded insights into the genetic structure of China indigenous pigs, but has focused on a limited number of populations. Here, we present an analysis of population structure and signals of positive selection in 42 Chinese pig populations that represent the most extensive pig phenotypic diversity in China, using genotype data of 1.1 million SNPs on customized Beadchips. This unravels the fine‐scale genetic diversity, phylogenic relationships, and population structure of these populations, which shows remarkably concordance between genetic clusters and geography with few exceptions. We also reveal the genetic contribution to North Chinese pigs from European modern pigs. Furthermore, we identify possible targets of selection in the Tibetan pig, including the well‐characterized hypoxia gene (*EPAS1*) and several previously unrecognized candidates. Intriguingly, the selected haplotype in the *EPAS1* gene is associated with higher hemoglobin contents in Tibetan pigs, which is different from the protective role of *EPAS1* in the high‐altitude adaptation in Tibetan dogs and their owners. Additionally, we present evidence for the causality between *EDNRB* variants and the two‐end‐black (TEB) coat color phenotype in all Chinese pig populations except the Jinhua pig. We hypothesize that distinct targets have been independently selected for the formation of the TEB phenotype in Chinese pigs of different geographic origins. This highlights the importance of characterizing population‐specific genetic determinants for heritable phenotype in diverse pig populations.

## 1 INTRODUCTION

The domestication of farm animals is a groundbreaking event that has deeply influenced human history. The pig (*Sus scrofa*) was domesticated largely in China and the Near East approximately 10,000 years ago (Frantz et al., 2015; Larson et al., 2005). Since then, human‐mediated artificial selection and natural selection have resulted in diverse pig breeds in the world. Chinese domestic pigs represent a rich genetic resource. More than one‐third (~100) of global pig breeds are found in China (Wang et al., 2011). These diverse breeds are renowned for desirable traits related to reproduction, disease resistance, docility, and meat quality, providing valuable germplasm that supports the sustainable development of the pig industry not only in China but also in Western countries (Wang et al., 2011). For instance, both historical documents and genomic analyses have shown that Chinese aboriginal pigs have significantly contributed to the formation of European modern breeds such as Large White via a human‐mediated introgression during the onset of Industrial Revolution (Bosse et al., 2014; Chen et al., 2019).

With the implementation of the reform and opening‐up policy, China has witnessed unprecedented development of the pig industry over the past four decades. It is undeniable that an important part of this development has been built on continuous importation of European modern breeds (mainly Large White, Landrace, and Duroc). These modern breeds have experienced intensive breeding for lean pork production since the end of World War II and are dominating Chinese pig industry because of their excellent performance in growth rate, lean meat percentage, and feed conversion efficiency. The extensive use of European breeds in the past decades presents a great threat to Chinese indigenous pigs. Due to the preferential use of European modern breeds and the indiscriminate hybridization between European and Chinese breeds, the population sizes of the majority of Chinese local breeds have reduced dramatically over the past decades, of which at least 20 breeds are extinct now (Wang et al., 2011). To solve this alarming problem, Chinese government launched a national conservation program for 42 indigenous pig breeds, a representative of Chinese pig germplasm. This program financially supports the in situ conservation of a nucleus herd comprising at least 10 consanguineously unrelated boars and 100 sows for each of the 42 breeds in a well‐managed farm (Wang et al., 2011). Farm owners are required to establish a reliable strategy to maintain genetic diversity to the utmost by avoiding severe inbreeding within each breed, which needs an in‐depth investigation of population structure and phylogenetic relationships of these breeds and possible admixture events among these breeds.

Although a number of studies have been conducted to improve our understanding of population genetics of Chinese local breeds, one limitation of these previous studies lies in the fact that they are based on sparse markers (mitochondria DNA and microsatellites) or a limited number of breeds (Chen et al., 2018; Diao et al., 2019; Xu et al., 2019). The tremendous development of genomics has driven the transition of sparse marker‐based studies to high‐density marker‐dependent researches in pigs (Groenen, 2016; Yang et al., 2017). In this study, by using a customized DNA chip containing 1.1 million SNPs, we provide the most comprehensive genomic analysis of Chinese local pigs from 42 diverse breeds, covering the majority (*n* = 36) of the 42 breeds listed in the Chinese national conservation program for livestock genetic resources. This work uncovers the current status of genetic diversity of Chinese indigenous pigs, unravels the fine‐scale phylogenetic relationships, population structure, historical admixture, and split of Chinese local pigs in a context of global populations.

Tibetan pigs are geographically distinct from other Chinese breeds, which have evolved excellent adaptation to the harsh environment in the Qinghai‐Tibetan plateau (Wang et al., 2011). We and other researchers have explored the 60K SNP and genomic sequence data of a limited number of individuals to identify candidate loci for high‐altitude adaptation in Tibetan pigs (Ai et al., 2014; Li et al., 2013). Here we used the 1.1 million SNP data from the 42 breeds to detect genomic loci putatively under selection for the plateau adaptability, which advances our understanding of the molecular mechanisms of the hypoxic adaptation in Tibetan pigs. Moreover, several Central Chinese breeds and Jinhua pigs (Table S1) have the so‐called “two‐end‐black” (TEB) coat color phenotype, which is characterized by a white body with black heads and hips (Wang et al., 2011). Previous investigations indicate that *EDNRB* is a candidate gene for this interesting phenotype (Ai, Huang, & Ren, 2013; Wang et al., 2015; Wilkinson et al., 2013). In this study, the chip SNP data enabled us to obtain the compelling evidence that *EDNRB* is the gene responsible for the TEB phenotype and to identify a candidate causative mutation at the *EDNRB* locus for this phenotype in Chinese pigs.

## 2 MATERIALS AND METHODS

### 2.1 Sample collection and DNA extraction

**Figure 1:**
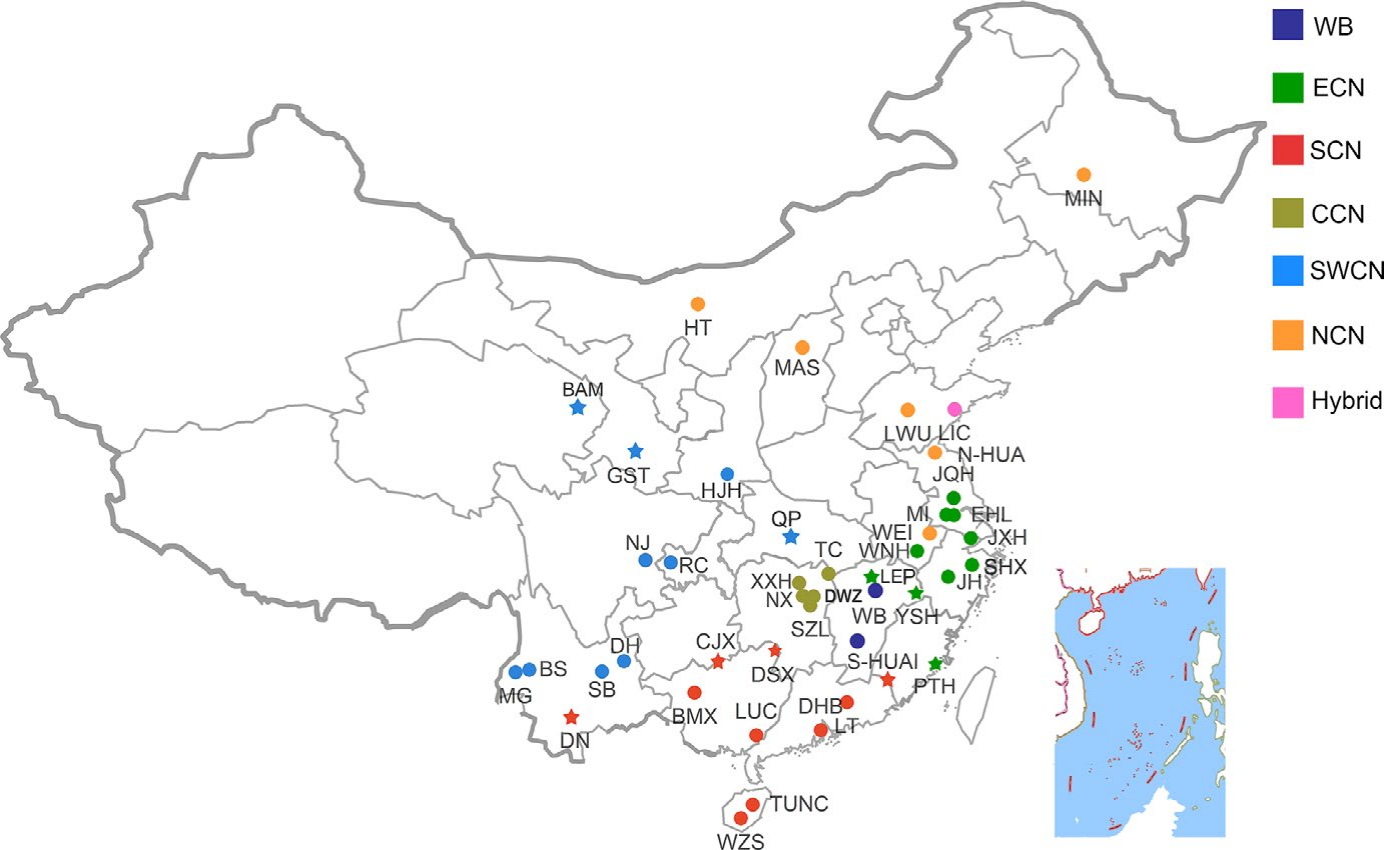
Geographical location of pig breeds sampled in this study. Abbreviations for these breeds and their ecotypes are given in Table 1. Each dot represents one breed. Different colors denote different ecotypes. Nine breeds having an admixture with their neighboring ecotype lineages are indicated by asterisks. Wild boars were sampled from multiple regions in Central China

All procedures used for this study and involving animals were in compliance with guidelines for the care and utility of experimental animals established by the Ministry of Agriculture of China. A total of 718 pigs from 42 Chinese indigenous breeds (Table S1), one Chinese synthetic breed (Sutai), two Chinese wild boar populations, and four European breeds were used in this study (Table 1, Figure 1). Chinese indigenous pigs except for Tibetan pigs were sampled from nucleus herds in state‐owned conservation farms at 41 localities around China. European pig samples were collected from two commercial companies in Jiangxi province. These pigs were genetically unrelated and had no common ancestor within three generations according to their pedigree. Tibetan pigs were sampled from four villages at altitude of 3,200 m in Hezuo county, Gansu province. Genomic DNA was extracted from ear tissues of these pigs using a routine phenol/chloroform protocol and was diluted to a final concentration of 100 ng/μl. The integrity of genomic DNA was verified with agarose gel electrophoresis.

**Table 1:** Samples and their genomic diversity statistics

Breed	Abbrev^a^	Origin	Ecotype	No.	*N*_snp_ b	$P_{N}$ c	$H_{o}$ d	π e	$r_{0.3}^{2}$(kb)^f^
Chinese pigs									
Erhualian	EHL	Wuxi, Jiangsu	ECN	15	706,396	0.69	0.22	0.23	22.53
Jinhua	JH	Jinhua, Zhejiang	ECN	15	579,283	0.57	0.19	0.19	46.34
Jiangquhai	JQH	Taizhou, Jiangsu	ECN	17	673,930	0.66	0.22	0.21	36.10
Jiaxinghei	JXH	Jiaxing, Zhejiang	ECN	15	446,347	0.44	0.15	0.16	160.73
Lepinghua	LEP	Leping, Jiangxi	ECN	15	779,871	0.77	0.23	0.24	25.26
Mi	MI	Jintan, Jiangsu	ECN	15	555,469	0.55	0.19	0.18	46.44
Putianhei	PTH	Putian, Fujian	ECN	15	759,239	0.75	0.23	0.24	25.54
Shengxianhua	SHX	Shengxian, Zhejiang	ECN	15	730,131	0.72	0.21	0.23	24.79
Wannanhua	WNH	Wannan, Anhui	ECN	15	739,545	0.73	0.23	0.24	17.00
Yushanhei	YSH	Yushan, Jiangxi	ECN	15	755,333	0.74	0.24	0.24	17.85
Bamaxiang	BMX	Bama, Guangxi	SCN	15	686,959	0.68	0.21	0.23	24.46
Congjiangxinag	CJX	Congjiang, Guizhou	SCN	15	647,202	0.64	0.20	0.22	37.01
Dahuabai	DHB	Xingfeng, Guangdong	SCN	15	656,012	0.65	0.22	0.22	44.42
Diannan	DN	Dehong, Yunnan	SCN	15	845,070	0.83	0.23	0.27	10.80
Dongshanxiang	DSX	Quanzhou, Guangxi	SCN	15	663,112	0.65	0.21	0.22	27.09
Lantang	LT	Heyuan, Guangdong	SCN	15	677,088	0.67	0.22	0.22	27.53
Luchuan	LUC	Luchuan, Guangxi	SCN	15	651,926	0.64	0.20	0.21	26.39
Huai (Fujian)	S‐HUAI	Longyan, Fujian	SCN	15	712,552	0.70	0.21	0.22	27.22
Tunchang	TUNC	Tunchang, Hainan	SCN	15	793,669	0.78	0.22	0.24	16.97
Wuzhishan	WZS	Wuzhishan, Hainan	SCN	15	866,526	0.85	0.25	0.28	9.39
Daweizi	DWZ	Changsha, Hunan	CCN	15	842,014	0.83	0.23	0.27	10.80
Ningxiang	NX	Ningxiang, Hunan	CCN	15	775,095	0.76	0.23	0.25	15.14
Saziling	SZL	Xiangtan, Hunan	CCN	15	833,079	0.82	0.23	0.27	9.44
Tongcheng	TC	Tongcheng, Hubei	CCN	19	769,237	0.76	0.23	0.25	14.11
Xiangxihei	XXH	Yuanjiang, Hunan	CCN	15	823,924	0.81	0.26	0.28	21.36
Bamei	BAM	Huzhu, Shannxi	SWCN	15	808,656	0.80	0.24	0.26	17.66
Baoshan	BS	Baoshan, Yunnan	SWCN	9	824,475	0.81	0.27	0.29	9.87
Dahe	DH	Fuyuan, Yunnan	SWCN	10	781,705	0.77	0.25	0.26	11.79
Tibetan (Gansu)	GST	Hezuo, Gansu	SWCN	17	885,966	0.87	0.24	0.27	11.56
Hangjianghei	HJH	Hanzhong, Shaanxi	SWCN	15	775,749	0.76	0.26	0.25	14.26
Mingguang Xiaoer	MG	Tengchong, Yunnan	SWCN	13	892,523	0.88	0.26	0.29	8.73
Neijiang	NJ	Neijiang, Sichuan	SWCN	9	634,626	0.62	0.20	0.22	24.75
Qingping	QP	Dangyang, Hubei	SWCN	15	866,684	0.85	0.26	0.27	12.95
Rongchang	RC	Rongchang, Chongqing	SWCN	15	746,372	0.73	0.23	0.25	13.34
Saba	SB	Chuxiong, Yunnan	SWCN	15	792,654	0.78	0.26	0.27	10.37
Hetao Daer	HT	Wuyuan, Inner Mongolia	NCN	15	854,014	0.84	0.28	0.28	13.08
Laiwu	LWU	Laiwu, Shandong	NCN	15	774,599	0.76	0.24	0.25	22.61
Mashen	MAS	Datong, Shanxi	NCN	15	699,126	0.69	0.24	0.23	28.31
Min	MIN	Lanxi, Heilongjiang	NCN	15	789,547	0.78	0.26	0.27	21.40
Huai (Jiangsu)	N‐HUAI	Donghai, Jiangsu	NCN	15	757,704	0.74	0.25	0.24	22.45
Wei	WEI	Xuancheng, Anhui	NCN	15	748,846	0.74	0.24	0.24	28.88
Lichahei	LIC	Jiaohe, Shandong	Hybrid	15	907,509	0.89	0.30	0.32	14.43
Sutai	SUT	Suzhou, Jiangsu	Hybrid	15	861,324	0.85	0.28	0.29	21.56
Wild boar	WB	Jiangxi	—	14	807,875	0.79	0.24	0.27	5.76
European pigs									
Duroc	DU	U.S.A	—	15	611,487	0.60	0.18	0.19	62.79
Landrace	LR	Denmark	—	15	736,241	0.72	0.23	0.24	34.20
Large White	LW	U.K	—	15	680,676	0.67	0.23	0.23	53.56
Pietrain	PI	Belgium	—	16	680,833	0.67	0.22	0.22	49.38

Abbreviations: CCN, Central China; ECN, East China; NCN, North China; SCN, South China, SWCN, Southwest China.

a Abbreviations of breeds.

b The number of qualified SNPs after filtering process.

c The proportion of polymorphic markers.

d Observed heterozygosity.

e Nucleotide diversity.

f Linkage disequilibrium values of *r* ^2^ = .3.

### 2.2 Customization of porcine 1.4M SNP chips

A customized Axiom Pig1.4M array plate (Affymetrix) was designed based on whole‐genome sequence data of 188 pigs including 150 Chinese indigenous pigs from 19 breeds and 38 European commercial pigs and wild boars following a series of criterion proposed by the manufacturer (Affymetrix). Briefly, the coding SNPs were preferentially selected, all SNPs were segregating in at least half of the 150 Chinese local pigs and were uniformly distributed on the genome, and the minor allele frequency (MAF) of each SNP was greater than 0.05 in both Chinese and European pigs. A set of 1,364,568 SNPs were explored to produce the 1.4M SNP Beadchips.

### 2.3 SNP genotyping and data filtering

First, 652 individuals were genotyped for 1,364,568 SNPs on the customized porcine 1.4M SNP Beadchips according to the manufacturer protocol (Affymetrix). Then, 15,762 unsuccessfully genotyped SNPs, 163,725 disconcordant SNPs among three duplicate samples, and 69,450 unmapped SNPs were discarded from further analyses, leaving 1,115,629 informative SNPs for these 652 individuals with call rates of greater 0.9. The average physical distance between adjacent SNPs was 2,689 bp. Next, we called genotypes of these 1,115,629 SNPs for additional 66 Chinese and European pigs from their whole‐genome resequencing data (Ai et al., 2015; Zhu et al., 2017). The 66 pigs included six Bamei, 10 Tibetan (Gansu), six Hetao Daer, six Jinhua, six Min, four Tongcheng, six Wuzhishan, 15 Large White pigs, and six Chinese wild boars. Nine Chinese pigs that showed obvious signatures of admixture with European pigs were discarded from further analyses (see below). A final set of 1,115,629 SNPs of 709 pigs from 48 Chinese and European breeds and wild boars (hereafter referred as to 1.1M SNP data set) were used for all subsequent statistical analyses unless otherwise specified.

### 2.4 Genetic diversity statistics

We removed SNPs with minor allele frequencies of less than 0.05 and individual call rates of less than 0.9 in the 1.1M SNP data set. A common subset of 1,018,478 SNPs (hereafter referred as to 1.0M SNP data set) was explored to calculate three parameters of genetic diversity including nucleotide diversity (π), the proportion of polymorphic markers ($P_{N}$), and observed heterozygosity ($H_{o}$) using vcftools (for π) and PLINK v1.9 (for $P_{N}$, $H_{o}$) (Chang et al., 2015).

### 2.5 Phylogenetic analysis

**Figure 2:**
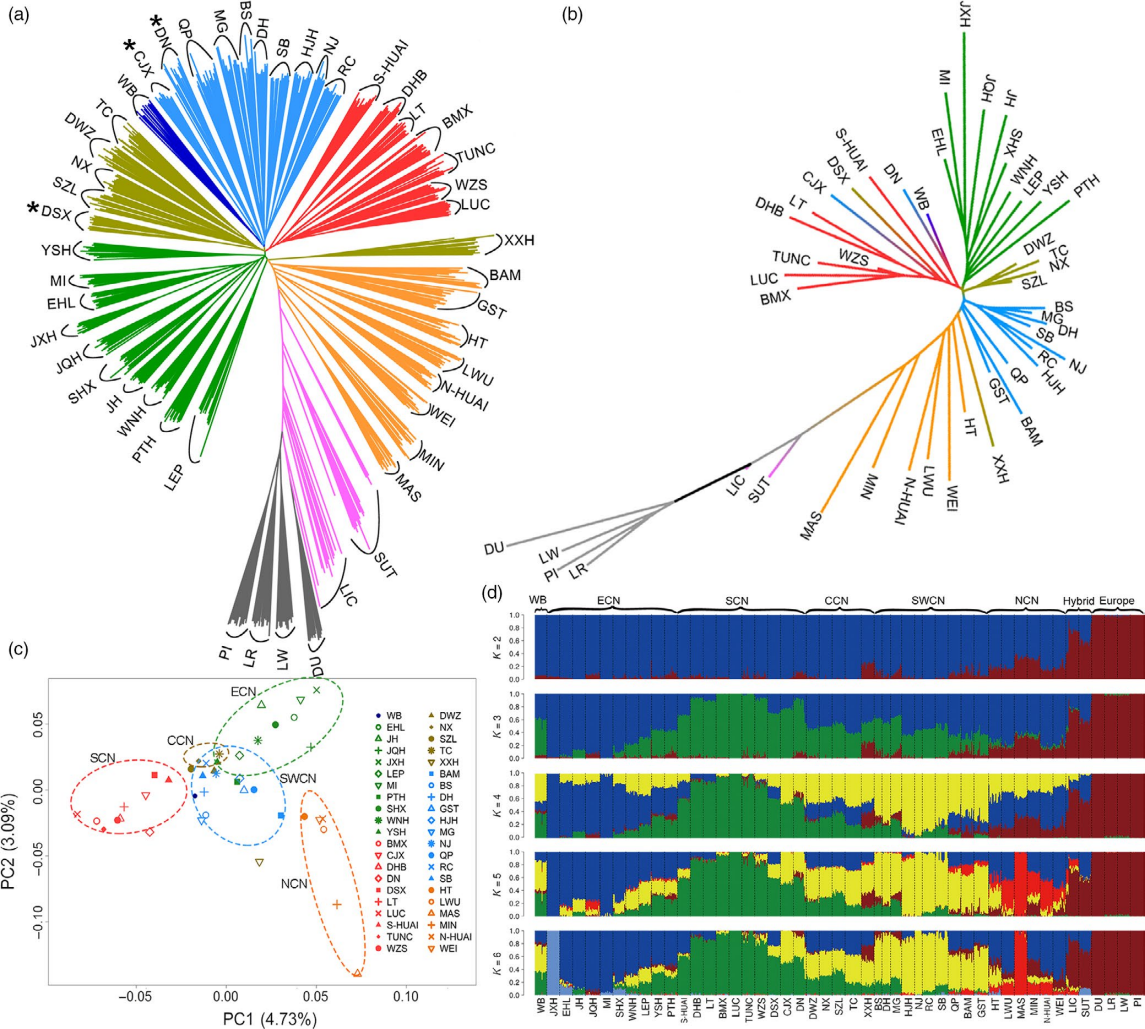
Phylogenetic relationships and population structure of Chinese indigenous pigs. (a) Neighbor‐joining (NJ) phylogenetic tree of 709 pigs tested in this study. The NJ tree was constructed using pairwise identical‐by‐state values among these 709 individuals (Materials and Methods). Three breeds (CJX, DN, and DSX) that were classified into different ecotypes in a $F_{\text{ST}}$‐based phylogenetic tree (b) are indicated by asterisks. (b) NJ phylogenetic tree of 47 Chinese and Europe domestic breeds and wild boars. The NJ tree was constructed by pairwise $F_{\text{ST}}$ values among these breeds (Materials and Methods). (c) Principal component plot of Chinese breeds and wild boars. Each figured point represents the average eigenvector of one breed. The first (PC1) and second components (PC2) are shown. Color codes for different ecotypes are as in Figure 1. (d) Population structures were inferred using ADMIXTURE with the assuming number of ancestral cluster *K* from 2 to 6. Each color represents one ancestral cluster and each vertical line represents one pig. The length of the colored segment in each vertical indicates the individual estimated fractional membership for each cluster. Breeds are separated by black dotted lines. Abbreviations for breeds and their ecotypes are given in Table 1

Genetic distances between individuals and breeds were calculated using an identity‐by‐state (IBS) similarity matrix and an average pairwise $F_{\text{ST}}$ matrix via PLINK (Chang et al., 2015). Neighbor‐joining (NJ) phylogenetic trees based on the calculated genetic distance were first constructed for the 718 pigs (Figure S1) and 48 breeds using PHYLIP version 3.5 (Felsenstein, 1989) and were visualized using FIGTREE (Rambaut, 2016). Nine pigs in the NJ tree including five Dahe, two Mingguang, and two wild boars were located at intermediate positions between Chinese and European pigs (Figure S1), which was most likely resulted from admixture events. These nine pigs were hence discarded for further analyses. NJ trees were then generated for the remaining 709 pigs (Figure 2a) and the corresponding 48 breeds (Figure 1) as mentioned above.

### 2.6 Inference of population differentiation and structure

Principal component analysis (PCA) was performed by GCTA v1.26 (Yang, Lee, Goddard, & Visscher, 2011), which first generated genetics matrix using “—make‐grm” option and then calculated the first four principal components with “—pca4” option. PCA plots were drawn using in‐house scripts and the R language. Heat maps of average pairwise $F_{\text{ST}}$ values between breeds were generated using the R language. Pairwise Nei standard distance between breeds were calculated using the following formula (Nei & Roychoudhury, 1974):

$$D=-\ln\frac{\sum_{i}\sum_{i}x_{\mathrm{ij}}y_{\mathrm{ij}}}{\sqrt{\sum_{i}\sum_{j}x_{\mathrm{ij}}^{2}\sum_{i}\sum_{j}y_{\mathrm{ij}}^{2}}},$$

where $i=1,2,\ldots ,r,j=1,2,\ldots ,k_{i}.$ $k_{i}$ is the number of alleles at allele *i*. The admixture analysis was conducted using ADMIXTURE (Tang, Peng, Wang, & Risch, 2005). During this analysis, the 1.1M SNP data set was further pruned using the “–indep‐pairwise 50 10 0.1” command in PLINK v1.9 (Chang et al., 2015). Then, the pruned data set was used to calculate Q values that reflect the maximum likelihood estimation of individual ancestries via ADMIXTURE. Bar plots of ancestry compositions of the tested breeds and wild boars were depicted using the R language with the assuming number of ancestors from 2 to 6.

### 2.7 Running of homozygosity and LD decay analyses

Running of homozygosity (ROH) value of each individual was determined using the “‐‐homozyg ‐‐homozyg‐window‐snp 50 ‐‐homozyg‐window‐het 1 ‐‐homozyg‐window‐missing 5 ‐‐homozyg‐kb 500” command in PLINK v1.9 (Chang et al., 2015). Then, the number of ROH was classified into three categories: 0.5–1 Mb (*R*_0.5–1 Mb_), 1–5 Mb (*R*_1–15 Mb)_), and >5 Mb (*R*_>5 Mb_). For the LD decay analysis, we used a subset of SNPs that had minor allele frequencies of greater than 0.1 and call rate more than 0.9 within each breed. Then, the genotype correlation coefficients ($r^{2}$) were calculated for all SNP pairs using the “–r2 –ld‐window‐kb 1000 –ld‐window‐r2 0.3” command in PLINK v1.9 (Chang et al., 2015). The LD extent and decline within each breed was predicted by formula described in previous studies (Heifetz et al., 2005; Sved, 1971).

### 2.8 Genome‐Wide scans for selection signals

We explored the locus‐specific branch length (LSBL) analysis (Shriver et al., 2004) to uncover genomic regions possibly under selection in Chinese indigenous pigs. Locus‐specific branch length x for population A between populations B and C were calculated using pairwise $F_{\text{st}}$, where *x* = ($F_{\text{stAB}}+F_{\text{stAC}}-F_{\text{stBC}}$)/2 and A, B, and C are the three populations under consideration. To identify genetic variants associated with local adaptation of Tibetan pigs, we defined three contrasting populations for the LSBL analysis. Population A included all Tibetan (Gansu) pigs. Population B comprised all individuals of nine breeds from South China including Bamaxiang, Congjiangxiang, Dahuabai, Dongshanxiang, Lantang, Luchuan, Huai (Fujian), Tunchuang, and Wuzhishan. Population C contained all individuals of 10 breeds from East China including Erhualian, Jinhua, Jiangquhai, Jiaxinghei, Leping Spotted, Mi, Putianhei, Shengxianhua, Wannanhua, and Yushanhei. To uncover genomic signals of positive selection for the two‐end‐black (TEB) coat color phenotype, we performed comparisons among another three populations: (a) population A included five TEB‐colored breeds (Bamaxiang, Luchuan, Dongshanxiang, Tongcheng, and Saziling); (b) population B comprised five breeds from East China with a solid black coat color phenotype (Erhualian, Jiaxinghei, Jiangquhai, Mi, and Shengxianhua); (c) population C included 11 breeds from South and Southwest China with a solid black or black body coat color phenotype (Tunchang, Lantang, Wuzhishan, Bamei, Baoshan, Dahe, Tibetan (Gansu), Mingguang, Neijiang, Qingping, and Saba). LSBL scores were calculated for all qualified SNPs in the 1.0M SNP data set using in‐house R language scripts as previously reported (Ai et al., 2014; Shriver et al., 2004). For the LSBL analysis of Tibetan pigs, 67 genes that harbored or were less than 10 kb away from SNPs with LSBL scores of greater than 0.8 were defined as candidate genes for high‐altitude adaptation. ClueGO implemented in Cytoscape (Bindea et al., 2009) was used to conduct the enrichment analysis of Gene Ontology (GO) terms and KEGG pathways for these 67 candidate genes.

### 2.9 Analysis of *EPAS1* and *EDNRB* haplotypes

A 38.4‐kb region (SSC3: 100,173,669 – 100,212,129 bp) encompassing 26 SNPs and the complete *EPAS1* gene was phased using PHASE (Stephens, Smith, & Donnelly, 2004), and the phased haplotypes were used to construct a haplotype network using pegas in the R package (Paradis, 2010). To test the association between the *EPAS1* haplotypes and hematologic traits, we collected blood samples from 54 Tibetan (Gansu) pigs of 1–2 years old and measured hematologic parameters using a CD1700 Whole Blood Analyzer (Abbott). We selected seven tag SNPs representing all *EPAS1* haplotypes in Tibetan (Gansu) pigs and genotyped these tag SNPs on these 54 Tibetan (Gansu) pigs by Sanger sequencing using primers listed in Table S2. The association test was conducted to uncover the relationship between *EPAS1* haplotypes and hematologic traits. We first tested the homogeneity of variance using *F*‐test and then evaluated the significance of *EPAS1* haplotypes using *t* test in the R language.

A 165.2‐kb region (SSC11: 54,611,837 – 54,777,019 bp) harboring 89 SNPs and the entire *EDNRB* gene was phased using BEAGLE (Browning & Browning, 2009). The phased haplotypes were explored to construct a haplotype‐sharing heat map using the pheatmap package in the R language.

### 2.10 RNA sequencing (RNA‐seq)

Black and white skin tissues were collected from Bamaxiang piglets raised in Jiangxi Lvfeng Pig Breeding Company and Jinhua piglets in Zhejiang Qinglian Food Company after slaughter. In addition, white skin tissues were harvested from DLY (Duroc X (Landrace X Large White)) hybrid pigs in a local abattoir. The skin tissues were stored in RNAlater (SIGMA) solution at −80°C after removing the dermal layer fat. RNA was extracted using the Trizol reagent (Invitrogen). Genomic DNA was isolated using a routine phenol/chloroform method. cDNA was prepared using the PrimeScript RT reagent Kit with gDNA Eraser (TaKaRa).

Skin RNA samples of Bamaxiang (four black and four white skin tissues) and Jinhua (four black and four white skin tissues) pigs with RIN value (integrity number) of greater than 6.8 were sequenced via Novogene Company. cDNA libraries were constructed and sequenced by pair‐end of 150 bp on a HiSeq 4000 sequencer (Illumina) platform. The clean data were first aligned to the pig reference genome (*Sscrofa* 11.1) using STAR v2.5.3a (Dobin et al., 2013). The StringTie v1.3.3 (Pertea et al., 2015) and featureCounts (Liao, Smyth, & Shi, 2014) software were then explored to calculate the counts of annotated genes. DESEQ2 (Love, Huber, & Anders, 2014) was finally used to identify differentially expressed genes (DEGs) between black and white skin. Functional enrichment analyses of DEGs were conducted via ClueGO (Bindea et al., 2009). We used IGV v2.4.5 (Thorvaldsdottir, Robinson, & Mesirov, 2013) to visualize RNA‐seq data in target regions.

### 2.11 Isoform Sequencing (Iso‐seq)

Three skin RNA samples each from Bamaxiang, Jinhua, and DLY pigs with RIN values of >6.5 were used to construct SMRTbell sequencing libraries via a Sequel platform (Pacbio). Three SMRTbell libraries were constructed for each sample. The library sizes were 1–4 kb and 4–10 kb. One SMRT cell sequencing reaction was performed for each library, which generated 15 Gigabyte (Gb) in total. The raw data were processed and analyzed according to the following procedure. Circular consensus sequences (CCSs) were first obtained using the default parameter of the ccs method in SMRTLink v5.1 (https://www.pacb.com/). The default parameters of the pbtranscript classify method in SMRTLink v5.1 were further used to determine the full‐length transcripts (full‐length reads) from CCSs. We then clustered and polished subreads using the ICE (Iterative Clustering and Error) algorithm implemented in the pbtranscript cluster method in SMRTLink v5.1. High‐quality consensus sequences were aligned to the pig reference genome (*Sscrofa* 11.1) via the parameters of “‐‐min‐trimmed‐coverage 0.9 ‐‐min‐identity 0.85” in the GMAP software (Wu & Watanabe, 2005). We used the parameter (‐i 0.9 ‐c 0.85) of collapse_isoforms_by_sam.py in TOFU (Gordon et al., 2015) to remove redundant sequences and aligned the removed redundant data to the pig reference genome (*Sscrofa* 11.1) via the above‐mentioned parameters in GMAP (Wu & Watanabe, 2005). The default parameters of the matchAnnot.py method in the MatchAnnot software (https://github.com/TomSkelly/MatchAnnot) were finally explored to annotate the transcripts. The annotated transcripts that were not mapped to the reference genome were considered as new transcripts.

### 2.12 RT‐PCR and qPCR

The *EDNRB* transcripts in the skin of Bamaxiang, Jinhua, and DLY pigs were amplified using the specific primers and optimal annealing temperatures (Table S3 **)**. PCR products were visualized by 1% agarose gel electrophoresis and were Sanger sequenced to verify their identities. To quantify the relative contents of *EDNRB* alternative transcripts in skin tissues of Bamaxiang, Jinhua, and DLY pigs, reverse transcription quantitative PCR (RT‐qPCR) was performed using SYBR Premix Ex Taq II kit (TaKaRa). The 10 μl reaction included 25 ng of cDNA, 0.4 μl of primers, and 0.2 μl of ROX. The F1/R1 and F3/R3 primers (Table S3) were used to amplify the normal and alternative transcripts, respectively. *GAPDH* was used as the internal reference gene. The expression level of the alternative transcript in relation to the normal transcript was calculated using $2^{-\Delta \Delta C_{t}}$ (Livak & Schmittgen, 2001). $\Delta \Delta C_{t}=\left(C_{t_{\text{ins}}}-C_{t_{\text{GAPDH}}}\right)-C_{t_{\text{ref}}}$, where $C_{t_{\text{ins}}}$, $C_{t_{\text{GAPDH}}}$ and $C_{t_{\text{ref}}}$ indicate *C*_t_ values of the alternative transcript.

### 2.13 Detection of *EDNRB* variants

Primers F4/R4 and F5/R5 (Table S3) were used to amplify genomic DNA of 167 Landrace, 233 Large White, 173 Duroc, 143 DLY pigs, 77 Pingxiang two‐end‐black pigs, and 93 Dongxiang spotted pigs. PCR products were sequenced to determine the genotypes of the *EDNRB* causal mutation (Table S3). Publicly available whole‐genome sequence data of 288 pigs representing 26 global breeds were further explored to detect variants in the *EDNRB* genes. The BWA software (Li & Durbin, 2009) was used to align the sequence data to the pig reference genome (*Sscrofa* 11.1). The SNPs and Indels of the *EDNRB* were identified using the default parameters of Platypus v0.8.1 (http://www.well.ox.ac.uk/platypus).

## 3 RESULTS

### 3.1 Genetic diversity of Chinese indigenous pigs

We sampled 709 genetically unrelated pigs from four European modern breeds, one European × Chinese hybrid breed, 42 Chinese indigenous breeds across China, and one Chinese wild boar population (Figure 1, Table 1). These pigs were successfully genotyped for a total of 1,115,629 SNPs on a customized SNP chip. We calculated nucleotide diversity (π), the number of polymorphic makers ($N_{\text{SNP}}$), the proportion of polymorphic makers ($P_{N}$), observed heterozygosity ($H_{O}$) within each breed using the chip SNP data. The ranges of these four statistics were 446,347 to 907,509 ($N_{\text{SNP}}$), 0.44 to 0.89 ($P_{N}$), 0.18 to 0.30 ($H_{o}$), and 0.16 to 0.32 (π), respectively. The largest values were observed in Lichahei pigs (907,509, 0.89, 0.30, and 0.32), followed by Mingguangxiaoer pigs (892,523, 0.88, 0.26, and 0.29), while the smallest values were evidenced in Jiaxinghei pigs (446,347, 0.44, 0.15, and 0.16), followed by Jiangquhai pigs (673,930, 0.66, 0.22, and 0.21) (Table 1). In general, Chinese indigenous pigs had more abundant genetic diversity than European commercial pigs, as the majority of Chinese indigenous breeds had larger (*p* < .05) $N_{\text{SNP}}$, $P_{N}$, $H_{o},$ and π values than European modern breeds. However, we noticed that three breeds from East China (Jinhua, Jiaxinghei, and Mi) displayed smaller (*p* < .05) values of these four statistics than all four European modern breeds (Table 1), reflecting reduced genetic variability in the three East Chinese breeds.

### 3.2 Phylogenetic relationships of Chinese indigenous pigs

To examine phylogenetic relationships of the 709 pigs, we constructed a neighbor‐joining (NJ) tree for these pigs based on an identical‐by‐state (IBS) distance matrix (Figure 2a). All individuals from the same breed formed their own clusters, indicating that these breeds may have undergone different evolutionary scenario due to regional adaption selection or genetic drift after domestication. The NJ tree revealed a clear divergence between European modern breeds and Chinese indigenous breeds, as the four European modern breeds (Large White, Landrace, Duroc, and Pietrain) formed a separate clustered and the majority of Chinese local breeds defined a large grouping. The Chinese breeds in general clustered according to their geographic origins and formed four major subgroupings: (i) pigs from East China, (ii) pigs from South China, (iii) pigs from Central China, and (IV) pigs from Southwest China. All six breeds from North China (Min, Mashen, Wei, Huai, Laiwu, and Hetao), two Southwest Chinese breeds (Tibetan Gansu and Bamei), and one Central Chinese breed (Xiangxihei) deviated from the large grouping encompassing Chinese breeds. However, these nine breeds appeared to be more genetically close to other Chinese breeds than to European breeds. Sutai, a European × Chinese hybrid breed, showed an intermediate phylogenetic relationship with Chinese and European breeds. Chinese Lichahei pigs are known to have an admixture of European breeds (Wang et al., 2011) and displayed the same clustering pattern with Sutai pigs (Figure 2a).

Next, we constructed a NJ tree for 47 breeds and Chinese wild boars comprising the 709 pigs using pairwise genetic differentiation ($F_{\text{ST}}$) values among these breeds. In agreement with the clustering results from the IBS distance matrix, European breeds showed a remarkable genetic differentiation from Chinese breeds by forming a distinct clade in the NJ tree. Among the 41 Chinese breeds, geographical neighbors always clustered together (Figure 2b) except for Xiangxihei: a most likely admixed breed (see below). Breeds from East China, South China, Southwest China, and Central China defined a monophyletic group, respectively. The six breeds from North China and the Xiangxihei from Central China clustered with other Chinese breeds in a paraphyletic pattern, forming a major clade that was separated from the Europe clade. Two hybrid breeds, Sutai and Lichahei pigs, clustered with the four European breeds with relatively long branch lengths (Figure 2b). The phylogenetic relationships among the tested European and Chinese breeds were also visualized by a heat map of pairwise Nei genetic distances among these breeds (Figure S2). The map showed larger genetic distances between the European and Chinese breeds in comparison with those among Chinese breeds. The four breeds from an adjacent region of Central China (Daweizi, Ningxiang, Shaziling, and Tongcheng, Figure 1) were more closely related to each other than to other Chinese breeds. Among the five groupings of Chinese breeds, Central Chinese breeds and Southwest Chinese breeds were more closely related (Figure S2).

We further conducted a PCA to uncover population differentiation among the breeds tested in this study. PC1 distinguished European modern breeds from Chinese indigenous breeds, and PC2 revealed genetic differentiation among Chinese breeds, especially between East Chinese breeds and South Chinese breeds (Figure S3). The two hybrid breeds (Sutai and Lichahei) were located at intermediate positions between Chinese and European clusters (Figure S3). When we excluded the four European breeds and the two hybrid breeds, the PCA analysis illustrated five major groupings formed by the 41 Chinese breeds, corresponding to their five geographical origins: East China, South China, Southwest China, Central China, and North China (Figure 2c).

### 3.3 Population structure of Chinese indigenous pigs

To investigate evolutionary origin and historical admixture patterns of all tested breeds, we performed the ADMIXTURE analysis (Tang et al., 2005) for these breeds assuming ancestral number *K* from 2 to 6 (Figure 2d). When *K* = 2, two basal lineages represented European and Chinese pigs, respectively. The two hybrid breeds (Sutai and Lichahei) were inferred as a mixture of these two divergent lineages. The six breeds from North China (Min, Mashen, Hetaoxiaoer, Laiwu, Huai, and Wei) and one Central Chinese breed (Xiangxihei) also showed clear evidence of admixture with European breeds. A small fraction of European lineages was evidenced in a number of Chinese breeds. From *K* = 3–4, three ancestral lineages were inferred in Chinese breeds: One was highly enriched in Jinhua and Mi pigs from East China, one in Bamaxiang, Luchuan, and Tunchang from South China, and the other in Neijiang, Rongchang, and Hanjianghei from Southwest China. The other breeds were admixed descendants of these three genetic components. Within the three major groupings of Chinese breeds (South China, East China, and Southwest China), each breed displayed a major component of its own ancestral lineages. Central Chinese breeds exhibited an equally admixed pattern of the three lineages, while North Chinese breeds did not show gene flow from South Chinese breeds but had an ancestral composition of East Chinese, Southwest Chinese, and European lineages. The ancestral composition pattern of all breeds remained unchanged when *K* = 6 except that one East China breed (Jiaxinghei) and one North Chinese breed (Mashen) formed an independent lineage, respectively (Figure 2d).

**Figure 3:**
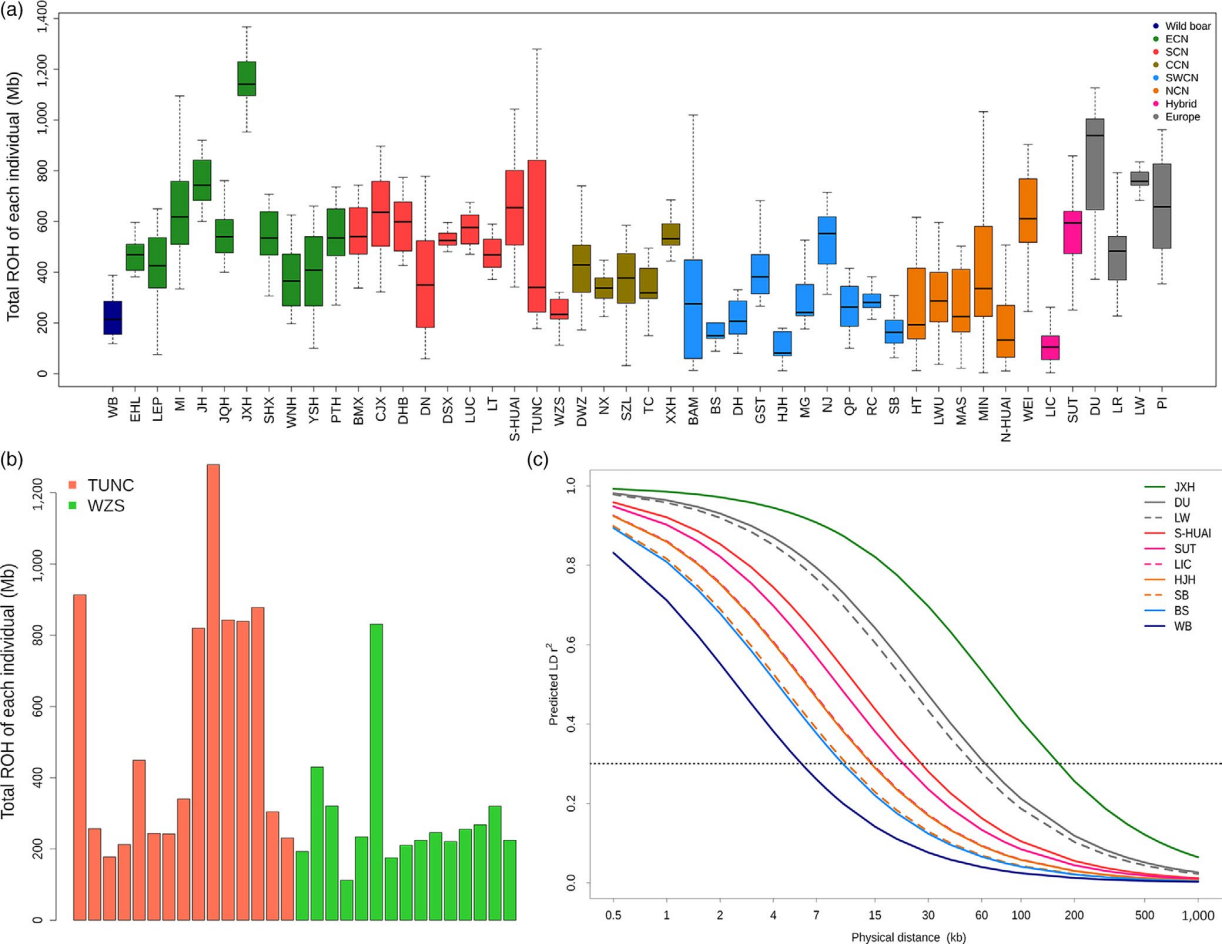
Running of homozygosity (ROH) and linkage disequilibrium (LD) decay. (a) The total ROH values of each individual in 47 Chinese and European domestic breeds and wild boars tested in this study. (b) The ROH values of each individual in Tunchang (TUNC) and Wuzhishan (WZS) pigs. (c) LD decay of four breeds (Saba, Hanjianghei, Baoshan and Lichahei) with the lowest and five breeds (Jiaxinghei, Duroc, Large White, Jinhua and Sutai) with highest ROH values. Wild boars are shown as an outgroup to domestic breeds. Abbreviations for breeds and their ecotypes are given in Table 1

To assess the inbreeding level within each breed, we calculated runs of homozygosity (ROH) using 1.1 million biallelic SNPs (Figure 3a). In agreement with previous studies (Ai et al., 2015, 2013; Bosse et al., 2012), the four European modern breeds in general had longer runs of homozygosity than Chinese aboriginal breeds. One exception is that Jiaxinghei pigs showed a lager ROH value than all four European breeds, an indicative of a high inbreeding coefficient in this breed. Three Southwest Chinese breeds (Baoshan, Hanjianghei and Saba) and one North Chinese breed (Huai) displayed roughly comparable ROH values to that of Chinese wild boars. We noted that ROH values varied considerably within several Chinese breeds like Bamei, Diannanxiaoer, and Tunchang, especially Tunchang (Figure 3a). Tunchang and Wuzhishan pigs are geographical neighbors and both are originally distributed in Hainan Island (Figure 1). All Wuzhishan individuals (*n* = 15) had roughly comparable and low ROH values except for one individual with a much longer ROH. In contrast, six of 15 Tunchang pigs showed remarkably longer ROH than the other nine individuals (Figure 3b), an indicative of a high inbreeding level and an inappropriate management of this breed. Interestingly, Sutai pigs, a hybrid breed, had much longer ROH than another hybrid breed (Lichahei, Figure 3a, Table S4 **)**. This is likely due to the fact that Sutai is a recently developed breed from a hybrid between Chinese Erhualian/Meishan and European Duroc pigs and has experienced selective breeding for less than 30 years (Wang et al., 2011), resulting in longer ROH. According to historical documents, Lichahei pigs had an admixture with European breeds (mainly Berkshire) approximate 100 years ago (Wang et al., 2011). The longer admixture history accumulated more recombination events to form shorter ROH in Lichahei pigs in comparison with Sutai pigs.

We further evaluated linkage disequilibrium (LD) extents in all tested breeds and wild boars by estimating their $r_{0.3}^{2}$ values, the physical distance at which the pairwise genotypic association in the 1.1M SNP data set decays below a threshold of 0.3. The LD extents were in general longer in European breeds than Chinese breeds (Table 1, Figure 3c), which is consistent with precious reports (Ai et al., 2015, 2013). Among the four European breeds, Landrace had the shortest LD extent ($r_{0.3}^{2}=34.2$ kb), which was nevertheless longer than those of 36 Chinese breeds (Table 1). Breeds with large ROH values usually showed long LD extents and vice versa (Figures 3c and S4). Of note, Jiaxinghei pigs displayed a longer LD extent even than the four European modern breeds that have experienced intensively selective breeding over the past decays. This provides further evidence that the Jiaxinghei breed is highly inbred.

### 3.4 Genomic regions under selection for high‐altitude adaptation

**Figure 4:**
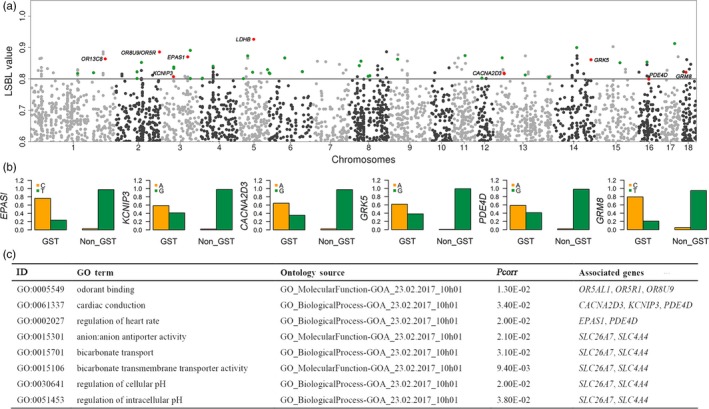
Genetic differentiation between Tibetan (Gansu) and non‐Tibetan pigs. (a) Manhattan plot of locus‐specific branch length (LSBL) values that were calculated using a three‐population‐contrasting model (Materials and Methods). Sixty‐seven genes (Table S5) that harbor or are less than 10 kb away from SNPs with LSBL values of greater than 0.8 are indicated in green. Ten genes that have apparent function related to hypoxia adaptation are highlighted in red. (b) Allele frequencies of top SNPs at six candidate genes for high‐altitude adaptation between Tibetan pigs in Gansu province (GST) and non‐Tibetan pigs. (C) GO terms and KEGG pathways in which 67 candidate genes for high‐altitude adaptation highlighted by the LSBL analysis are enriched

Tibetan pigs are renowned for their adaptability to the adverse living conditions of the Qinghai‐Tibetan Plateau, the roof of the world. We conducted the LSBL analysis to search for signatures of selection for high‐altitude adaptation across the genome of Tibetan pigs in Gansu Province using a three group‐contrasting model (see Materials and Methods). We identified 164 SNPs with LSBL values of greater than 0.80 at an empirical threshold of 0.02% (Figure 4a, Table S5). These SNPs showed remarkable genetic differentiation and allelic imbalance between Tibetan pigs and non‐Tibetan pigs (Figure 4b, Table S5) and are located within or 10 kb upstream or downstream of 67 uniquely annotated genes (Table S5) on the *Sscrofa* 10.2 genome assembly (http://www.ensembl.org/Sus_scrofa/Info/Index). The 67 genes were statistically enriched in several GO terms that are functionally related to high‐altitude adaptation, such as heart contraction, cardiac conduction, regulation of blood circulation, and regulation of intracellular pH (Figure 4c). Among these genes, several stood out to promising candidates for high‐altitude adaptation in Tibetan pigs, including *EPAS1*, *CACNA2D3*, *PDE4D*, *OR13CB*, *OR8U9*, *OR5R*, *GRM8*, *LDHB, SLC4A4,* and *SLC26A7* (Figure 4a). *EPAS1* is a well‐recognized gene for plateau adaptability in multiple species (Gorkhali et al., 2016; Huerta‐Sanchez et al., 2014; Miao, Wang, & Li, 2017). *CACNA2D3*, *KCNIP3,* and *PDE4D* are functionally related to blood circulation and heart contraction (Figure 4c). Beneficial alleles within these genes could have been selected to speed up blood flow for oxygen delivery in Tibetan pigs, compensating the effect of low oxygen concentration. *OR13CB*, *OR8U9*, *OR5R1,* and *OR5AL1* are four olfaction receptor genes. Tibetan pigs are raised in a cage‐free grazing way. We thus assume that natural selection on these four genes may enable Tibetan pigs to more efficiently look for food. *GRM8* encodes a glutamate metabotropic receptor that is a major excitatory (A. C. Chen et al., 2009; Scherer, Soder, Duvoisin, Huizenga, & Tsui, 1997). The receptor plays a critical role in memory and cognitive function (Elia et al., 2011). The signature of selection in these genes may contribute to the establishment of quickly response to attract under grazing conditions in Tibetan pigs. *LDHB* encodes the B subunit of lactate dehydrogenase enzyme that catalyzes the interconversion of pyruvate and lactate with concomitant interconversion of NADH and NAD^+^ during glycolysis (Gaspar et al., 2007). It is widely expressed in multiple tissues and highly in heart. The expression level of *LDHB* increases in response to myocardial infarction (Feng et al., 2019; Le et al., 2010). Variants in this gene could have been preferentially selected to fully utilize glycogen as an energy source in case of oxygen shortage and anaerobic glycolysis, a critical ecological factor restricting the viability of highland animals. In addition, *SLC26A7* is a member of the sulfate/anion transporter genes and is known to regulate intracellular pH through chloride channels (Kim, Shcheynikov, Wang, & Muallem, 2005). *SLC4A4* encodes a sodium bicarbonate co‐transporter (NBC), which involves in the regulation of the bicarbonate secretion and absorption and intracellular pH (Dinour et al., 2004; Nordstrom, Andersson, & Akerman, 2019). *SLC26A7* and *SLC4A4* beneficial variants may enable Tibetan pigs to regulate intracellular pH caused by strong anaerobic glycolysis.

### 3.5 Association of *EPAS1* haplotypes with hematological traits in Tibetan pigs

**Figure 5:**
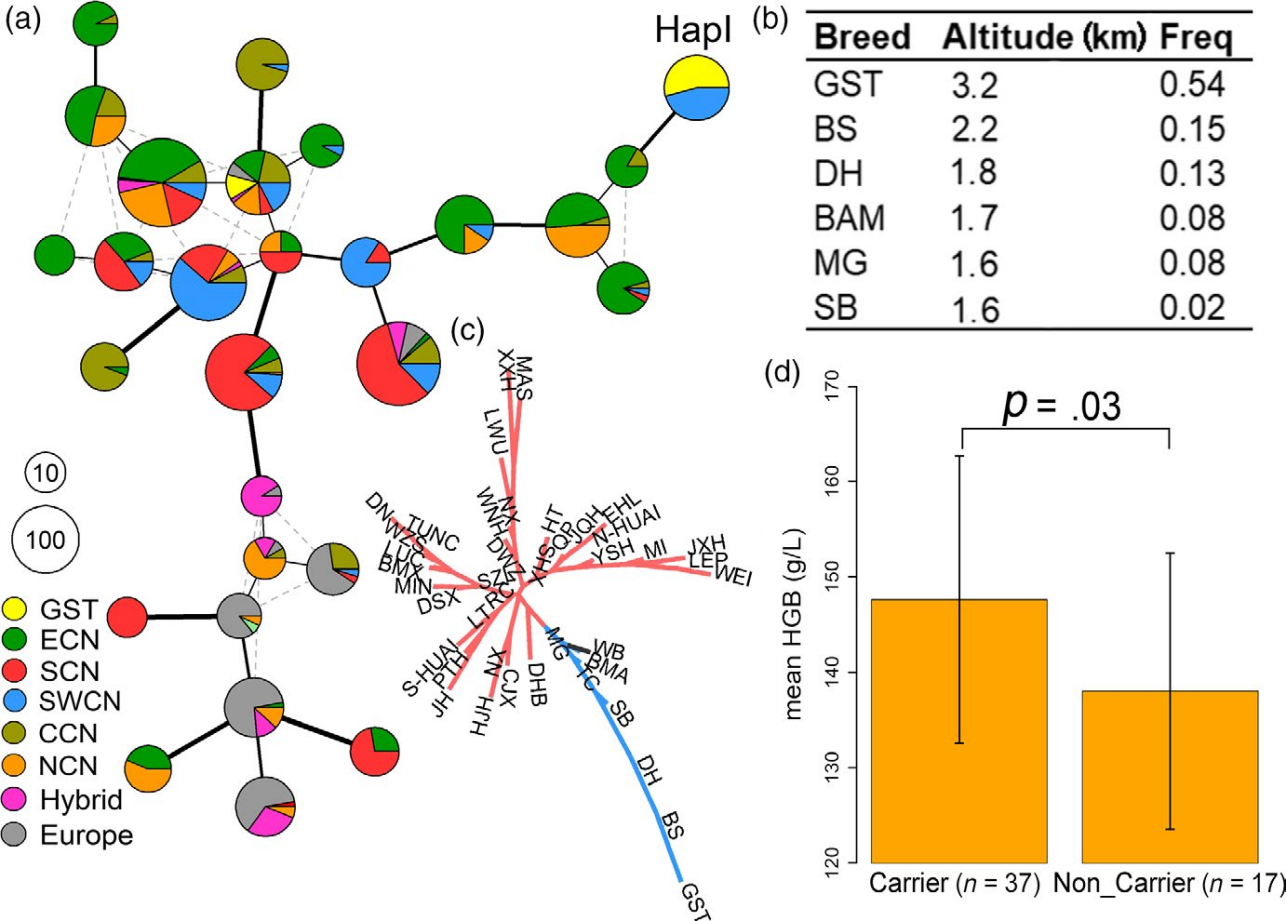
Selective signals at the *EPAS1* locus*.* (a) Network of *EPAS1* haplotypes with frequencies of greater than 10. The haplotypes were defined from 26 SNPs within the *EPAS1* region of 38.46 kb. Each pie chart represents one unique haplotype and the radius of pie chart is proportional to the number of each haplotype. The sections in the pie provide the breakdown of the haplotype representation among populations (ecotypes). The line width and length represent the variations between haplotypes. Different colors indicate different ecotypes. The major haplotype in Tibetan pigs in Gansu provicne (GST) is labeled by text HapI. Color codes for ecotypes are as in Figure 1. Abbreviations for breeds and their ecotypes are given in Table 1. (b) Frequencies of HapI in GST and other breeds. (c) Neighbor‐joining phylogenetic tree of 42 Chinese breeds and wild boars based on 26 SNPs in the *EPAS1* gene. (d) Association of HapI with the concentration of hemoglobin (HGB) in GST

*EPAS1* emerged as an extreme LSBL outlier (Table S5) in the genome scan of Tibetan and non‐Tibetan pigs. Considering the well‐established role of *EPAS1* in systemic response to hypoxia (Simonson, McClain, Jorde, & Prchal, 2012; Tian, McKnight, & Russell, 1997), we made a close examination in this gene. We first phased haplotypes of a 38.4‐kb region containing the complete *EPAS1* gene using 26 SNPs with this region and then built a haplotype network using 28 common haplotypes with a frequency of greater than 10 in Chinese pigs (Figure 5). We found that the most common haplotype in Tibetan (HapI, freq = 0.54) was slightly divergent from the other haplotypes (Figure 5a). This haplotype was only present in Tibetan pigs and five Southwest Chinese breeds (Bamei, Baoshan, Dahe, Mingguang, and Saba) that inhabit in maintain regions with an altitude of more than 1,600 meters (Figure 5b). We further constructed a phylogenetic tree of the 41 Chinese domestic breeds and wild boars using the 26 SNPs constituting the *EPAS1* haplotypes, which was distinct from the genome‐wide tree (Figure 2b). We observed a long evolutionary divergence between Tibetan pigs and low‐altitude breeds (Figure 5c). To explore the physiological relationship between the *EPAS1* haplotypes and the adaptation to hypoxia, we measured the hematologic parameters for 54 Tibetan adult pigs living in a farm with an elevation of 3,300 meters in Gansu Province. These pigs were genotyped for seven tag SNPs representing the *EPAS1* haplotypes using Sanger sequencing. We conducted association testing for the *EPAS1* haplotypes reconstructed from the seven tag SNPs and discovered a statistically significant association between the high‐altitude haplotype (HapI) and hemoglobin concentration. Individuals carrying this haplotype showed an increased hemoglobin concentration than noncarriers (147.65 ± 15.05 (g/L) versus 138.06 ± 14.46 (g/L), *p* = .03, Figure 5d, Table S6), which is a clear advantage in response to hypoxia in Tibetan pigs.

### 3.6 Selection signals for two‐end‐black coat color phenotypes in Chinese indigenous pigs

**Figure 6:**
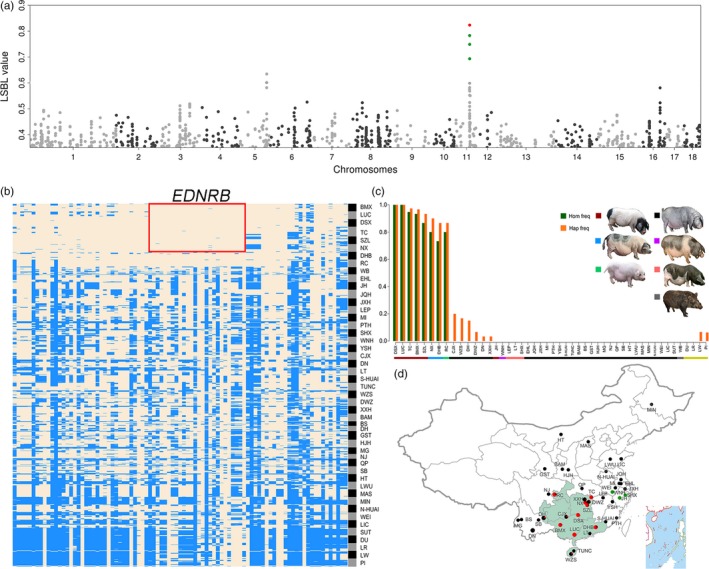
Selective signals at the *EDNRB* locus. (a) Manhattan plot of locus‐specific branch length (LSBL) values highlighting a strong selective signal for the two‐end‐black (TEB) coat color phenotype in Chinese indigenous pigs on chromosome 11. The LSBL values were calculated by a model contrasting breeds with the TEB phenotype against those without this phenotype (Materials and Methods). The red dot represents the top SNP with a LSBL value of 0.82 at 54,704,015 bp on chromosome 11. (b) Haplotypes at the *EDNRB* locus. The haplotypes were defined from 89 SNPs within a 165.2‐kb region harboring the *EDNRB* gene. The major allele of each SNP in TEB‐colored breeds (DSX, LUC, TC, BMX and SZL) is indicated in yellow and minor allele in blue. A total of 26 SNPs in a continuous region of 39.8 kb are nearly fixed in these TEB‐colored breeds. The 39.8‐kb region is highlighted by a red rectangular and perfectly corresponds to the *EDNRB* gene. (c) Frequencies of the 39.8‐kb *EDNRB* haplotype in Chinese and European pigs. Different colors in the horizontal bar indicate different coat color phenotypes of breeds. Green vertical lines indicate the frequencies of this haplotype in diverse breeds and orange lines represent the frequencies of individuals homozygous for this haplotype in diverse breeds. (d) Geographic distribution of Chinese breeds carrying the TEB‐associated *EDNRB* haplotype. Red dots represent breeds with this haplotype. Green dots denote breeds that show the TEB or white‐belted coat colors but lack the TEB‐associated *EDNRB* haplotype. Black dots indicate breeds with a solid black or black body with white belly coat color phenotype

To identify genomic regions that may have been targets of selection for the TEB phenotype, we calculated the LSBL values of 1.1 million SNPs by comparisons of genetic differentiation among three TEB‐colored and non‐TEB‐colored groupings (see Materials and Methods). We found that the four most extreme outlier SNPs clustered in a region of 92 kb on chromosome 11 with the top SNP at 54,704,015 bp on this chromosome (Figure 6a). We next turned our attention from the top signal to specific candidate genes. We phased haplotypes for a 165‐kb region harboring 89 SNPs including the top four SNPs in the 709 pigs. We noted that five TEB‐colored breeds (Bamaxiang, Dongshan, Luchuan, Shaziling, and Tongcheng) shared an identical haplotype of 40 kb comprising 26 SNPs, which were nearly fixed in these TEB‐colored breeds (Figure 6b). The 40‐kb region encompasses only one gene: *EDNRB*, a pigmentation gene that has been highlighted as a candidate for the TEB phenotype in previous studies(Ai et al., 2013; Wilkinson et al., 2013). Our data herein provide compelling evidence that *EDNRB* is a promising candidate for the TEB phenotype in Chinese indigenous pigs. We further investigated the distribution frequencies of the 40‐kb *EDNRB* haplotypes in the 48 Chinese and European breeds (Figure 6c). Homozygous carriers of the TEB‐associated haplotype were solely present in the five TEB‐colored breeds, one solid white breed (Rongchang), and two belted breeds (Dahuabai and Ningxiang) with black spots on the body. Heterozygous carriers of this haplotype were observed in five breeds from South and Central China (Congjiangxiang, Dahuabai, Diannan, Xiangxihei, and Wuzhishan) at a low frequency of less than 0.2. All breeds from North and East China did not have the TEB‐associated haplotype (Figure 6d). Intriguingly, Jinhua pigs, an East Chinese breed, also display the TEB phenotype but do not carry the TEB‐associated haplotype at the *EDNRB* locus (Figure 6c). It thus raises the possibility that the TEB phenotype is controlled by other distinct loci in Jinhua pigs.

### 3.7 RNA sequencing identified different DEGs between white and black skin of Bamaxiang and Jinhua pigs

**Figure 7:**
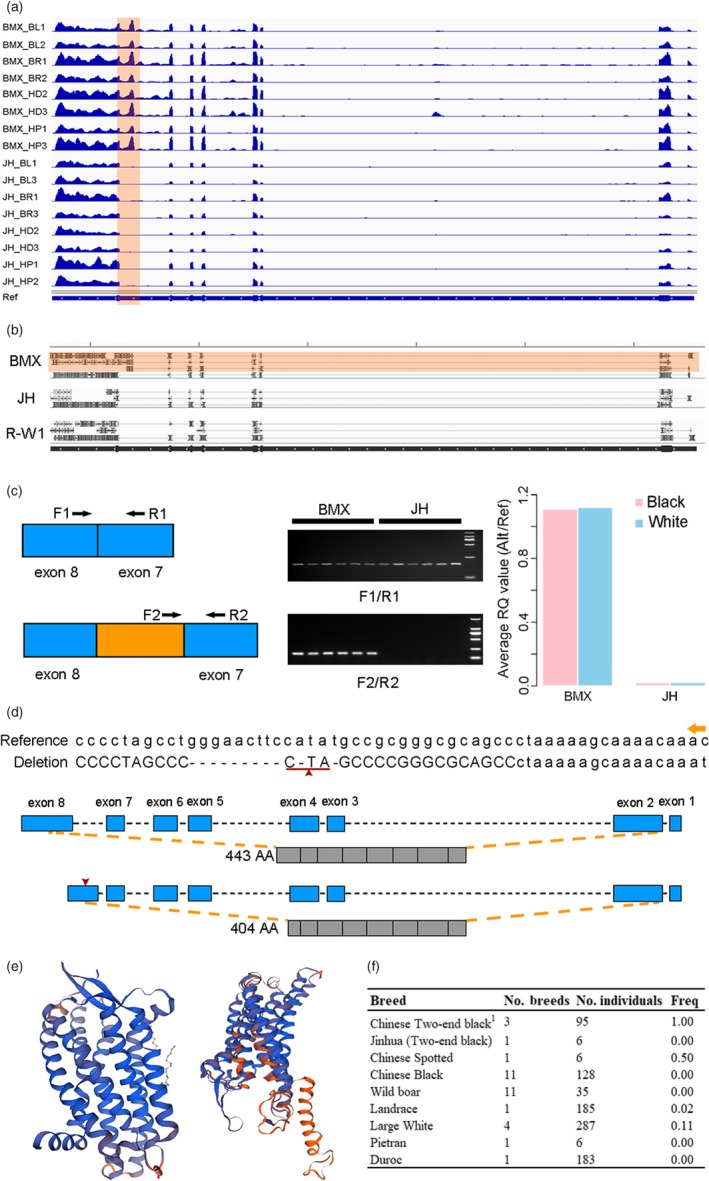
*EDNRB* transcripts and candidate causative mutation for the two‐end‐black coat color phenotype in pigs. (a) *EDNRB* transcripts in the skin of Bamaxiang and Jinhua pigs. RNA sequencing reads are aligned to the genomic sequence of *EDNRB* in the pig reference genome. The orange shaded region denotes alternative transcripts between Bamaxiang and Jinhua pigs. BMX_BL, Bamaxiang black skin; BMX_WH, Bamaxiang white skin; JH_BL, Jinhua black skin; JH_WH, Jinhua white skin (b) Whole‐length isoform transcripts in the skin of Bamaxiang (BMX), Jinhua (JH) and DLY pigs revealed by Iso‐seq. (c) RT‐PCR and RT‐qPCR of *EDNRB* transcripts in the skin of Bamaxiang (BMX) and Jinhua (JH) pigs. RT‐qPCR result is shown in the right panel and the y‐axis represents relative expression level (alternative splicing/reference). (d) Schematic diagram of *EDNRB* transcripts and their encoded protein. The DNA sequences (chromosome 11:50,076,938–50,076,994 bp) in the reference genome and Bamaxiang pigs are shown in the upper panel. The normal and alternative transcripts and their encoded proteins of *EDNRB* are shown in the middle and lower panels, respectively. The capital and lowercase represent exonic and intronic regions, respectively. The orange arrow indicates transcription direction (top). The red triangle or underline indicates the 11‐bp Indel that causes premature stop codon of the *EDNRB* alternative transcript in Bamaxiang pigs. (e) Predicted three‐dimensional protein structures of *EDNRB* encoded by the normal (left) and alternative (right) transcripts. (f) Allele frequencies of the 11‐bp deletion, the candidate causative mutation in the *EDNRB* gene (chromosome 11: 50,076,945–50,076,960 bp), in global pig breeds and wild boars. 1, three Chinese two‐end‐black breeds including Bamaxiang, Luchuan, and Pingxiang pigs

To uncover the molecular mechanism of the TEB phenotype, we further conducted RNA sequencing (RNA‐seq) on the skin tissues of Bamaxiang and Jinhua pigs. By comparing the RNA‐seq data of white skin and black skin, we only detected two differentially expressed genes (DEGs) (*p* < .01, $\log_{2}\left(\text{flod}\;\text{change}\right)>1$) between the two tissues in Bamaxiang pigs, including one up‐regulated gene (*TRPM1*) and one down‐regulated gene (ENSSSCG00000018923) in the black skin (Figure S5a). A total of 34 DEGs were identified in Jinhua pigs at the same significant threshold, of which six well‐characterized pigmentation genes (*DCT*, *MLANA*, *PMEL*, *SLC24A5*, *TYR,* and *TYRP1*) were up‐regulated in the black skin (Figures S5b,c). However, neither Bamaxiang pigs nor Jinhua pigs showed differences in the expression levels of *EDNRB*. We made a close examination on the RNA‐seq data using the IGV software and observed an extra exon between exon 7 and exon 8 of *EDNRB* in Bamaxiang pigs in comparison with the normal transcript in Jinhua pigs (Figure 7a).

### 3.8 Full‐length transcriptome analysis confirms the expression of alternative *EDNRB* transcripts in the skin of Bamaxiang pigs

To confirm the presence of alternative *EDNRB* transcripts in Bamaxiang pigs, we performed isoform sequencing (Iso‐seq) on three skin RNA samples each from Bamaxiang, Jinhua, and DLY pigs. Iso‐seq generated 275,719, 127,011, and 346,512 high‐quality CCS in Bamaxiang, Jinhua, and DLY pigs, respectively. After removing the redundant sequence, we aligned 117,945, 54,541, and 150,617 transcripts to the pig reference genome and identify 65,258, 32,244, and 79,282 annotated transcripts in Bamaxiang, Jinhua and DLY pigs, respectively. We did not find alternative transcripts in the skin tissues of Jinhua and DLY pigs at the *EDNRB* locus, while we observed both normal and alternative *EDNRB* transcripts in Bamaxiang pigs, which is consistent with the RNA‐seq result as visualized by the IGV software (Figure 7b). We further conducted RT‐PCR and RT‐qPCR analyses on these two *EDNRB* transcripts. RT‐PCR showed that the normal transcript was expressed in the skin tissues of Bamaxiang and Jinhua pigs, whereas the alternative transcript only existed in Bamaxiang pigs (Figure 7c). RT‐qPCR further unraveled that the expression level of the alternative transcript was nearly identical to that of the normal transcript in the black and white skin of Bamaxiang pigs, but the alternative transcript was hardly detected in Jinhua and DLY pigs (Figure7c). Further investigations are needed to clarify the reason for the expression of the two *EDNRB* transcripts in Bamaxiang pigs.

### 3.9 The causality of the *EDNRB* alternative transcript in relation to the TEB phenotype

*EDNRB* is a G‐protein‐coupled receptor that mediates signal transduction between cells by binding to three isoforms of EDN1, EDN2, and EDN3, and then acts on MITF to affect melanin synthesis (Opdecamp et al., 1997). *EDNRB‐*deficient mice display the TEB phenotype (Hosoda et al., 1994). We explored the online softwares Predictprotein (https://www.predictprotein.org/) and SWISS (https://swissmodel.expasy.org/) to predict the protein structure and three‐dimensional structure of *EDNRB* encoded by the normal and alternative transcripts, respectively. The normal transcript encodes 443 amino acids, and the alternative transcript encodes 404 amino acids due to the presence of a premature stop codon. The shorter peptide losses two helical transmembrane regions and four protein binding regions (Figure 7d) and had distinct three‐dimensional structures from the normal EDNRB protein (Figure 7e). We argue that this abnormal protein is likely to interfere with the binding of the normal EDNRB protein to its ligand, which in turn affects the synthesis of hair follicle melanin in the trunk and leads to the TEB phenotype ultimately.

We explored whole‐genome sequence data of 288 pigs from 26 global breeds and Sanger sequencing data of 160 pigs from two Chinese breeds (Pingxiang and Dongxianghua) to identify causative mutation in the *EDNRB* gene. We showed that all individuals from the three TEB‐colored breeds including Bamaxiang (*n* = 12), Luchuan (*n* = 6), and Pingxiang (*n* = 77) were homozygous for an 11‐bp deletion in the *EDNRB* coding region (SSC11: 50,076,945bp ‐ 50,076,960 bp). This deletion creates the premature stop codon in the *EDNRB* alternative transcript and thus causes a frameshift mutation, resulting in the truncated EDNRB protein (Figure 7d). We noted that the 11‐bp deletion homozygously existed only in Chinese TEB‐colored pigs (*n* = 95); was absent in Jinhua pigs (*n* = 6), Chinese black‐colored pigs (*n* = 128), European Duroc (*n* = 183), and Pietrain (*n* = 6); and was present in Chinese spotted pigs, Large White pigs and Landrace pigs at frequencies of 75% (134/178), 6% (33/574), and 1% (4/370), respectively (Figure 7f). The presence of the 11‐bp deletion in Large White pigs is likely due to the human‐mediated transportation of south Chinese pigs (Cantonese pigs with the TEB phenotype) into the England 200–300 years ago (Bosse et al., 2014), which contributed to the development of Large White and Landrace pigs. Altogether, we argue that the 11‐bp deletion in the *EDNRB* gene is a strong candidate causative mutation for the TEB coat color phenotype in Chinese pigs.

## 4 DISCUSSION

### 4.1 Phylogenetic classification and historical admixture of Chinese indigenous pigs

Chinese indigenous pigs have been traditionally classified into six ecotypes: South China, North China, Central China, Southwest China, the lower Yangtze River basin, and the Tibetan pigs (Figure 1). Here, our data support the Chinese indigenous pigs pertain to five groupings: South China, North China, Central China, Southwest China, and East China. First, Chinese local breeds were in general clustered together according to their geographic origins, forming the five groupings in both IBS‐ and $F_{\text{ST}}$‐derived NJ phylogenetic trees (Figure 2a,b). Second, the classification pattern of these five groupings was also evidenced by the PCA (Figure 2c). Notably, all North Chinese breeds including Hetao, Huai, Laiwu, Mashen, Min, and Wei have a clear signal of introgression with European modern breeds, as we observed (i) these six breeds deviated from the major grouping of Chinese local breeds in the IBS‐based NJ tree (Figure 2a); (ii) these breeds clustered with the other Chinese breeds in a paraphyletic manner, although the clustering formed a major clade separating from European pigs in the $F_{\text{ST}}$‐derived NJ tree (Figure 2b); (iii) these breeds have ancestral lineages of European breeds as revealed by the ADMIXTURE analysis (Figure 2d). Guirao‐Rico *et al*. (Guirao‐Rico, Ramirez, Ojeda, Amills, & Ramos‐Onsins, 2018) reported a putative recent and unidirectional parental gene flow from European pigs to Chinese pigs, which is consistent with our findings. Moreover, our results are in agreement with historical documents that European modern breeds were introduced into North China such as Hebei, Shandong, and Heilongjiang from the beginning to the mid of the last century (Wang et al., 2011). The introduced breeds were extensively explored to cross with local breeds to improve the meat production performance of these local breeds. The human‐mediated hybridization events explain why the genomes of North Chinese local breeds show the signature of admixture with European modern breeds and possess a mosaic of Chinese and European haplotypes. The admixture signature was also detected in Xiangxihei (Figure 2), a local breed from Central China.

It is certainly possible that gene flow between neighboring breeds may have taken place during the past thousands of years after pig domestication given the short distance between their origins. Here, these geographical neighbors usually have a close genetic relationship with each other and always clustered together in the NJ tree (Figure 2). It is thus conceivable that the heritage of neighboring ecotypes was evident in several Chinese breeds distributing in border regions of different ecotypes (Figure 1). For example, our phylogenetic analyses support the classification of Putianhei pigs into East Chinese breeds. The original habitat of Putianhei pigs is close to the distribution area of South Chinese breeds (Figure 1). As revealed by the ADMIXTURE analysis, Putianhei pigs have a proportion of South Chinese component (Figure 2d), displaying a considerable influence of South Chinese pigs. In contrast, the origin of Huai pigs from Fujian Province is fundamentally South Chinese; however, ADMIXTURE revealed a non‐negligible percentage of East Chinese germplasm in this South Chinese breed (Figure 2d). Furthermore, Wannanhua and Wei pigs are originally from the South area of Anhui Province (Figure 1), but are classified into East Chinese and North Chinese breeds, respectively. The East Chinese component is predominant in the two breeds. However, the Wananhua pig has a mixture of South Chinese lineage while the heritage of European breeds was introgressed into the Wei pig (Figure 3). This may cause the different classification results of the two breeds.

### 4.2 Distinct roles of *EPAS1* in plateau adaptability

To detect signature of adaption in Tibetan pigs, we used the LSBL statistic to identify alleles that have experienced remarkable changes in frequency in the Tibetan population relative to two reference populations (East China and South China). We detected strong signals of selection in 67 genes (Figure 4a and Table S5), of which *CCDC82*, *GPALPP1*, *KRT31*, *KRT34*, *LRRC2, SLC4A4,* and *SLC26A7* have not reported in previous studies. We note that three genes (*CACNA2D3*, *KCNIP3,* and *PDE4D*) play a role in heart contraction and blood regulation that are apparently related to plateau adaptability (Cantero‐Recasens et al., 2018; Kuhlenbaumer et al., 2006; van Rijn et al., 2005). The 67 genes also include several well‐characterized hypoxia genes including *EPAS1*. The *EPAS1* haplotype has been under preferential selection (Figure 4b) and is associated with an increase in the concentration of hemoglobin in Tibetan pigs (Figure 5d). It has been reported that Tibetan pigs have a higher concentration of hemoglobin and hence a higher capacity of delivering oxygen in the circulating blood than lowland pigs, which is thought to be an adaptive response to the hypoxic environment of the Tibetan plateau (Kong et al., 2014). Our data support that the elevated blood hemoglobin level could be in part due to the regulatory effect of the selected *EPAS1* haplotype in Tibetan pigs. Intriguingly, the adaptive role of *EPAS1* differs between Tibetan peoples and highland animals. The adaptive variants of *EPAS1* reduced hemoglobin concentrations in Tibetans relative to their lowland counterparts, serving as a crucial protection mechanism for excessive erythrocytosis in Tibetans (Beall et al., 2010; Yi et al., 2010). In Tibetan domestic dogs, the selected variations of *EPAS1* are also associated with lower blood flow resistance in high‐altitude populations (Gou et al., 2014). Here we show that the adaptive haplotype of *EPAS1* has a positive effect on rising blood hemoglobin contents in Tibetan pigs. These observations indicate that human and highland species have evolved distinct mechanisms for local adaptation to the inhospitable environments of the Tibetan plateau. Even the same gene, such as *EPAS1*, could have entirely different molecular mechanisms for plateau adaptability. It is known that the blood viscosity goes up with a rise in hemoglobin levels, increasing the risk of erythrocytosis and cardiac events. To avoid this risk, Tibetan pigs have evolved a distinguishable feature of blood parameters, that is, have lower mean corpuscular volume and erythrocyte aggregation indice to attenuate the side effect of increased hemoglobin levels (Teng et al., 2014). Future investigations are needed to characterize the genetic determinants underlying these phenotypic changes in Tibetan pigs.

### 4.3 Different genes are responsible for the TEB coat color phenotype in Chinese indigenous pigs of different origins

The TEB coat color phenotype is only found in Chinese indigenous pigs, including Bamaxiang, Luchuan, and Dongshan from South China, Pingxiang, Shaziling, and Tongcheng from Central China, and Jinhua from East China. *EDNRB* has been proposed as a candidate gene for this phenotype in previous studies (Ai et al., 2013; Wilkinson et al., 2013). Here we provide further evidence that *EDNRB* is the gene responsible for the TEB phenotype in Chinese indigenous pigs except Jinhua (Figure 6). The supporting evidence comes from the following observations: (i) the LSBL analysis identified the most significant signal within a 90‐kb region on chromosome 11 that harbors the top four extreme outlier SNPs and the *EDNRB* gene (Figure 6a); (ii) the haplotype‐sharing analysis uncovered an identical haplotype of 40 kb in all TEB‐colored breeds except Jinhua, and this haplotype perfectly corresponds to the *EDNRB* gene (Figure 6b); (iii) animals homozygous for the TEB‐associated *EDNRB* haplotype are found exclusively in TEB‐ or belted‐colored breeds except Jinhua (Figure 6c); and (iv) the TEB‐colored breed (Bamaxiang) express an alternative transcript that encodes a truncated EDNRB in the skin, contributing to the formation of the TEB phenotype. The alternative transcript is absent in the skin tissue of Jinhua pigs. (v) Except for Jinhua, all TEB‐colored breeds are homozygous for a 11‐bp deletion that creates the premature stop codon in the *EDNRB* alternative transcript. These results unexpectedly but clearly show that another gene(s) is responsible for the TEB phenotype in Jinhua pigs, which is worthwhile for further investigations. Notably, Luchuan and Jinhua pigs, two representatives of TEB‐colored breeds, represent two ancient lineages of Chinese breeds as revealed by the ADMIXTURE analysis (Figure 2d). The two breeds have most likely experienced different histories of artificial selection. Causative variants in *EDNRB* and another unknown gene could have been independently selected to form the same TEB coat color phenotype in Luchuan and Jinhua pigs of different geographical origins. This work highlights the importance of characterizing population‐specific genetic determinants for heritable phenotype and underscores the necessity of in‐depth investigation in diverse global pig populations.

## CONFLICT OF INTEREST

None of the authors have any competing interests in the manuscript.

## AUTHOR CONTRIBUTIONS

J.R. and L.H. designed the study and analyzed data. J.R. and M.H. wrote the paper. M.H., B.Y., and H.C. performed statistical analyses. H.Z., Z.W. and H.A. collected samples and phenotypic data and performed sequencing and genotyping experiments.

## Data Availability

The SNP genotype data used in this study are available at a publicly available repository (http://10.6084/m9.figshare.8282318), and the RNA sequence raw data are available from NCBI SRA under accession number PRJNA548997.
