# Supplementary material for: The fine‐scale genetic structure and selection signals of Chinese indigenous pigs
Source: Evol Appl. 2019 Nov 22;13(2):458–75. doi: 10.1111/eva.12887 (PMC6976964; doi:10.1111/eva.12887)
Supplement: Supplementary file 1 [file EVA-13-458-s001.pdf]

Supplementary Materials

Table S1 Location and characteristics of 42 Chinese pig breeds

See the "Table S1.xlsx" file

Table S2 Severn tag SNPs representing *EPAS1* haplotypes on chromosome 3

Position (bp)	Primer	Sequence (5'-3')
100184171	FP	CCCCCGGTGATAAGAAATGGG
	RP	GCCCAAAGGCTTCCAGAGTA
100198784	FP	CTGCATGGGTTGGAAATTGGG
	RP	AAGCTATCATGGACCCCTCG
100177923	FP	GATGCCCGCTAGGATGTTGA
	RP	GGTCTGCTGACTGCAAAAGC
100194326	FP	CCCTTAGCGGTGCTGGAATA
	RP	CTCTGCCGAACTTCCAGTCC
100212129	FP	ACGGAAATGGAATCCTGCTCA
	RP	CCAACCCATCTCATGGTGGT
100195554	FP	ATGCCTTTCCTTGAGGTCGG
	RP	CCCTGAACCGCCAGTTTTTG
100204652	FP	TCAGCTCTTGCCCCTTGATG
	RP	GGCTCCATGGCTTCTCAACT

Table S3 Primers for detecting *EDNRB* transcripts and mutations

**Primer**	**Sequence (5’-3’)**	**Annealing temperature**
F1/R1	F: GGAAGTTGTCATATCCGTGATCA<br>R: CAACATGGCGTCCCTGAATT	57 ℃
F2/R2	F: CTGTGGTGAAGGTTGCAGAC<br>R: CAACATGGCGTCCCTGAATT	
F3/R3	F: CAACATTCCCAACCCAGGGA<br>R: CACAGCAACACAGGATCCCA	
F4/R4	F: ACCCTGTCAAGGTCATTGAGTA<br>R: GACTGGCACCAGCAGCATAA	55 ℃
F5/R5	F: ACCCTGTCAAGGTCATTGAGTA<br>R: CAACATTCCCAACCCAGGGA	

Table S4 Genome-wide distribution of ROH per category

**Breed**	**ROH$_{(0.5\text{- Mb})}$**	**ROH$_{(1\text{-}5Mb)}$**	**ROH$_{(>5Mb)}$**
**Chinese pigs**			
EHL	2629	1849	148
JH	3063	2788	362
JQH	2795	2251	341
JXH	2656	3720	855
LEP	2213	1847	145
MI	3102	2594	279
PTH	2707	2481	202
SHX	2587	2407	274
WNH	2144	1609	169
YSH	2353	1784	122
BMX	2324	2127	267
CJX	1905	2095	408
DHB	2078	2230	321
DN	2079	1598	191
DSX	2817	2313	184
LT	2887	2463	108
LUC	3575	2601	149
S-HUAI	1808	2228	438
TUNC	2052	1830	320
WZS	1852	1045	120
DWZ	2351	1829	139
NX	2024	1413	144
SZL	2275	1934	96
TC	2549	1820	223
XXH	2438	2533	168
BAM	1853	910	183
BS	940	646	36
DH	1338	626	22
GST	2533	1697	1
HJH	1421	419	238
MG	1394	1064	93
NJ	1963	1565	99
QP	2038	1294	60
RC	2669	1414	34
SB	1623	703	24
HT	1657	946	118
LWU	3306	1476	20
MAS	3416	1082	2
MIN	2221	1312	220

N-HUAI	2251	909	6
WEI	2156	2108	389
LIC	1694	407	1
SUT	2403	2351	237
WB	949	829	125
**European pigs**			
DU	4678	3444	306
LR	4277	2284	62
LW	3277	2930	384
PI	4500	3158	204

Abbreviations for breeds are given in **Table 1**

Table S5 LSBL outlier SNPs and candidate genes for high-altitude adaptation in Tibetan pigs

Chr	Pos (bp)	SNP	LSBL	Nearest Gene	GST	Non_GST
1	28240674	C/T	0.83	-	-	-
1	34183450	C/T	0.86	-	-	-
1	39858637	C/T	0.87	-	-	-
1	39860028	C/T	0.83	-	-	-
1	39861663	C/T	0.82	-	-	-
1	39867697	A/G	0.83	-	-	-
1	39872404	C/T	0.84	-	-	-
1	39877999	A/G	0.82	-	-	-
1	39893382	C/T	0.81	-	-	-
1	62215449	A/G	0.83	*U6*	0.68/0.32	0.03/0.97
1	83750022	A/G	0.82	*AFG1L*	0.12/0.88	0.91/0.09
1	173275111	C/T	0.81	-	-	-
1	173321046	A/T	0.83	-	-	-
1	173352754	C/T	0.81	-	-	-
1	173402103	A/G	0.83	-	-	-
1	173405714	C/T	0.83	-	-	-
1	173409945	C/T	0.82	-	-	-
1	173415488	A/G	0.83	-	-	-
1	232103972	A/G	0.82	*NFIB*	0.56/0.44	1/0
1	266872281	C/T	0.82	-	-	-
1	266877123	C/G	0.81	-	-	-
1	267051772	A/G	0.88	-	-	-
1	267061082	A/G	0.89	-	-	-
1	275336992	G/T	0.86	*OR13C8*	0.38/0.62	0.99/0.01
2	20853630	C/T	0.82	-	-	-
2	20854387	A/G	0.81	-	-	-
2	76381742	A/G	0.82	*SGTA*	0.71/0.29	0.03/0.97
2	76386861	A/G	0.82	*SGTA*	0.29/0.71	0.97/0.03
2	76388696	C/T	0.82	*SGTA*	0.29/0.71	0.97/0.03
2	76392258	G/T	0.80	*SGTA*	0.29/0.71	0.96/0.04
2	76395903	A/G	0.82	*SGTA*	0.29/0.71	0.97/0.03
2	76397316	C/T	0.82	*SGTA*	0.29/0.71	0.97/0.03
2	92447894	A/G	0.83	*ATG10*	0.59/0.41	0.02/0.98
2	92575553	G/T	0.85	*ATG10*	0.41/0.59	0.01/0.99
2	92624312	A/T	0.80	-	-	-
2	133561349	A/G	0.81	-	-	-
2	159222004	C/T	0.89	*OR8U9*	0.71/0.29	0.02/0.98
3	48410423	A/G	0.81	*KCNIP3*	0.59/0.41	0.02/0.98
3	49121066	A/G	0.83	*TMEM127*	0.56/0.44	0.01/0.99

3	49137354	C/T	0.84	*STARD7*	0.53/0.47	0.01/0.99
3	90008005	A/C	0.81	-	-	-
3	93997195	A/G	0.80	-	-	-
3	100194326	C/T	0.87	*EPAS1*	0.76/0.24	0.03/0.97
3	100778801	C/T	0.81	-	-	-
3	109936299	C/T	0.80	*STRN*	0.32/0.68	0.97/0.03
3	110032611	C/T	0.89	*VIT*	0.79/0.21	0.02/0.98
3	110035493	A/G	0.80	*VIT*	0.15/0.85	0.92/0.08
4	8611777	A/C	0.80	*LRRC6*	0.38/0.62	0.97/0.03
4	9467234	A/T	0.80	-	-	-
4	49100995	A/C	0.84	*SLC26A7*	0.53/0.47	0.01/0.99
4	49129135	A/T	0.84	*SLC26A7*	0.5/0.5	0/1
4	49176545	T/G	0.80	*SLC26A7*	0.5/0.5	0.01/0.99
4	49178344	G/A	0.80	*SLC26A7*	0.5/0.5	0.01/0.99
4	49245313	A/G	0.80	*SLC26A7*	0.5/0.5	0.01/0.99
4	49250353	C/T	0.81	*SLC26A7*	0.53/0.47	0.01/0.99
4	49251859	G/T	0.80	*SLC26A7*	0.5/0.5	0.01/0.99
4	49252760	T/G	0.82	*SLC26A7*	0.53/0.47	0.01/0.99
4	49254953	G/T	0.80	*SLC26A7*	0.53/0.47	0.01/0.99
4	49257525	C/G	0.81	*SLC26A7*	0.53/0.47	0.01/0.99
4	49259030	A/G	0.81	*SLC26A7*	0.53/0.47	0.01/0.99
4	49260033	A/G	0.82	*SLC26A7*	0.53/0.47	0.01/0.99
4	49333306	A/G	0.80	-	-	-
4	63838740	A/G	0.81	-	-	-
5	17183757	C/T	0.80	*DIP2B*	0.41/0.59	0/1
5	17186069	A/G	0.80	*DIP2B*	0.41/0.59	0/1
5	27228173	A/C	0.81	-	-	-
5	27459439	C/G	0.83	-	-	-
5	27461626	C/T	0.83	-	-	-
5	33029968	C/T	0.87	*MSRB3*	0.76/0.24	0.03/0.97
5	33033862	C/T	0.87	*MSRB3*	0.76/0.24	0.03/0.97
5	51186517	C/T	0.82	*SSPN*	0.5/0.5	0.01/0.99
5	55086017	C/G	0.93	*LDHB*	0.29/0.71	1/0
5	81423158	A/G	0.82	-	-	-
5	105606669	A/G	0.83	*LIN7A*	0.5/0.5	0/1
6	269612	A/C	0.82	*TUBB3*	0.79/0.21	0.05/0.95
6	762440	A/G	0.82	*ANKRD11*	0.47/0.53	0.99/0.01
6	2758590	C/T	0.82	*FOXL1*	0.97/0.03	0.1/0.9
6	57647095	C/T	0.87	*A1BG*	0.21/0.79	0.97/0.03
7	19979173	C/T	0.86	-	-	-
7	50664320	C/T	0.85	-	-	-
7	100994873	A/G	0.85	-	-	-

7	114939308	C/T	0.82	-	-	-
7	125212302	A/G	0.83	-	-	-
8	32257261	A/G	0.81	*RFC1*	0.59/0.41	0.02/0.98
8	39477546	C/T	0.84	*ATP10D*	0.44/0.56	0.1/0.99
8	45569375	G/T	0.86	*CPE*	0.62/0.38	0.03/0.98
8	71942916	G/T	0.81	*SLC4A4*	0.71/0.29	0.04/0.96
8	78162657	C/T	0.81	*FRAS1*	0.44/0.56	0.99/0.01
8	107012111	C/G	0.83	-	-	-
8	107022462	C/T	0.81	-	-	-
8	107170414	A/G	0.84	-	-	-
8	107180581	C/T	0.83	-	-	-
8	123350734	A/G	0.83	-	-	-
8	139390404	C/T	0.80	-	-	-
8	139558905	G/T	0.89	-	-	-
9	32401369	C/T	0.86	*CCDC82*	0.38/0.62	0.99/0.01
9	33084004	C/T	0.88	-	-	-
9	48355486	A/G	0.83	-	-	-
9	67737873	A/G	0.86	-	-	-
9	67793610	C/T	0.82	-	-	-
9	67820560	A/C	0.81	-	-	-
10	15208746	A/C	0.82	-	-	-
10	67341243	A/G	0.84	-	-	-
11	9231108	C/T	0.85	-	-	-
11	17793048	C/T	0.84	-	-	-
11	22562982	A/G	0.87	*NUFIP1*	0.65/0.35	0.01/0.99
11	34936704	C/T	0.83	-	-	-
11	47420598	C/G	0.87	-	-	-
11	48876512	A/G	0.85	-	-	-
12	21402245	A/G	0.80	*KRT31*	0.26/0.74	0.96/0.04
12	37494370	A/C	0.80	-	-	-
13	32717854	G/T	0.87	*LRRC2*	0.12/0.88	0.95/0.05
13	35330688	C/T	0.83	-	-	-
13	38553459	A/T	0.82	-	-	-
13	40237328	A/G	0.82	*LRTM1*	0.65/0.35	0.02/0.98
13	117593410	A/G	0.81	*PHC3*	0.47/0.53	0/1
13	117595505	C/G	0.81	*PHC3*	0.47/0.53	0/1
13	117796930	G/T	0.81	-	-	-
13	117834685	A/G	0.87	-	-	-
13	128576882	C/T	0.82	-	-	-
13	128580711	A/G	0.82	-	-	-
13	200693380	C/T	0.81	-	-	-
13	204856043	C/T	0.80	*KRTAP7-1*	0.56/0.44	1/0

13	208450147	A/G	0.81	-	-	-
14	71119994	C/T	0.81	-	-	-
14	71125611	C/G	0.84	-	-	-
14	71345983	A/G	0.81	-	-	-
14	71347158	A/C	0.80	-	-	-
14	71350237	A/G	0.81	-	-	-
14	71351911	G/T	0.81	-	-	-
14	71353781	A/G	0.81	-	-	-
14	71355064	C/T	0.81	-	-	-
14	71360044	C/T	0.81	-	-	-
14	71361991	A/G	0.81	-	-	-
14	71363376	C/T	0.81	-	-	-
14	71366577	G/T	0.81	-	-	-
14	71368011	C/T	0.81	-	-	-
14	88230462	A/G	0.90	*ZMIZ1*	0.76/0.24	0.02/0.98
14	89216868	A/C	0.87	-	-	-
14	89226920	A/G	0.87	-	-	-
14	90322266	A/G	0.85	-	-	-
14	91202884	A/G	0.83	-	-	-
14	91215283	G/T	0.82	-	-	-
14	113948491	C/T	0.82	-	-	-
14	140706910	A/G	0.86	*GRK5*	0.62/0.38	0.01/0.99
15	68075988	G/T	0.90	-	-	-
15	93580077	C/T	0.85	*OSBPL6*	0.21/0.79	0.97/0.03
15	113838755	A/G	0.82	*U6*	0.44/0.56	0.99/0.01
15	113845055	C/T	0.83	-	-	-
16	5805256	A/C	0.82	-	-	-
16	35258764	A/C	0.81	-	-	-
16	35292898	G/T	0.85	*ARL15*	0.15/0.85	0.95/0.05
16	35295963	T/C	0.85	*ARL15*	0.15/0.85	0.95/0.05
16	41859629	A/G	0.81	*PDE4D*	0.95/0.41	0.02/0.98
16	41862681	A/G	0.81	*PDE4D*	0.95/0.41	0.02/0.98
17	22311428	G/T	0.83	-	-	-
17	50521474	C/T	0.91	*PTPRT*	0.79/0.21	0.02/0.98
17	62907460	G/T	0.87	-	-	-
17	62909714	A/G	0.87	-	-	-
18	13474698	G/T	0.82	-	-	-
18	22098383	C/T	0.82	*GRM8*	0.79/0.21	0.05/0.95
18	24936175	C/T	0.86	-	-	-
18	34572055	A/G	0.83	-	-	-
18	39621369	C/T	0.85	-	-	-

GST: Tibetan (Gansu) pigs; Non_GST: Non_Tibetan (Gansu) pigs

Table S6 Serum hemoglobin contents of 54 Gansu Tibetan pigs

Sample ID	HGB (g/L)	HapI haplotype carrier
A7	118	No
A8	151	No
B4	126	No
B6	135	No
B7	113	No
B9	166	No
C7	150	No
C9	138	No
C10	147	No
D3	136	No
D4	160	No
D6	125	No
D12	132	No
E4	149	No
E7	136	No
E8	125	No
E9	140	No
A1	122	Yes
A2	133	Yes
A3	144	Yes
A4	142	Yes
A5	141	Yes
A6	147	Yes
A12	148	Yes
B2	159	Yes
B3	134	Yes
B5	137	Yes
B8	137	Yes
B10	174	Yes
B11	145	Yes
B12	136	Yes
C1	139	Yes
C2	121	Yes
C3	170	Yes
C4	157	Yes
C5	160	Yes
C6	161	Yes
C8	157	Yes
C11	156	Yes

C12	177	Yes
D1	147	Yes
D2	180	Yes
D5	153	Yes
D7	156	Yes
D8	146	Yes
D9	166	Yes
D10	148	Yes
E1	115	Yes
E2	135	Yes
E6	139	Yes
E10	152	Yes
E11	153	Yes
E12	144	Yes
F1	132	Yes

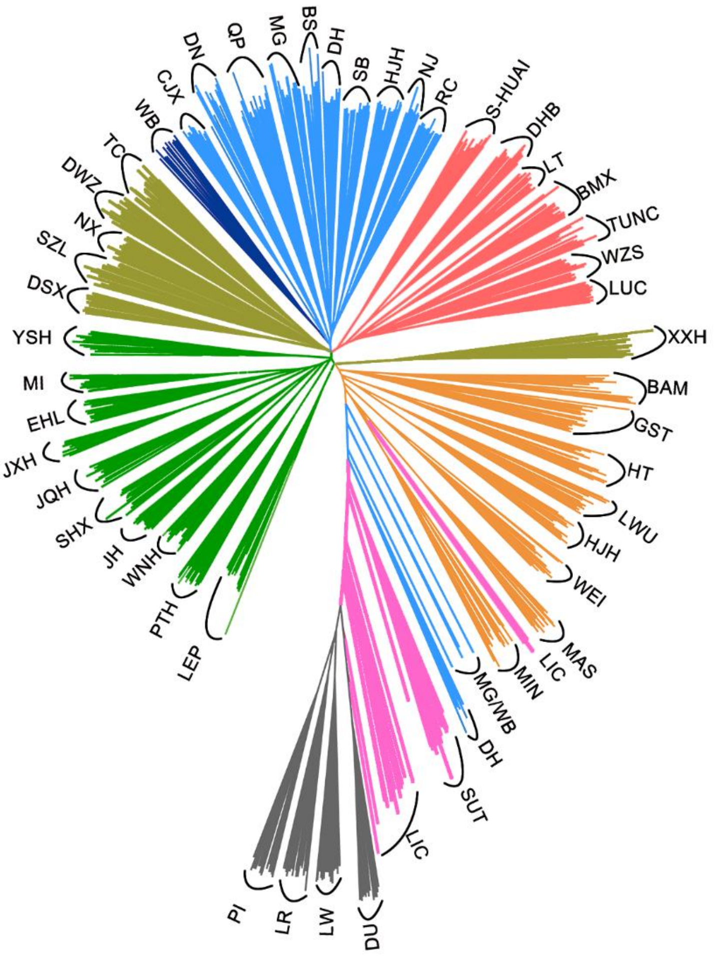

Figure S1 Neighbor-joining phylogenetic tree of 718 pigs tested in this study. In this tree, two MG pigs, two wild boars and five DH pigs locate at intermediate positions between Chinese and European pigs, which is likely resulted from admixture events. These nine pigs were hence discarded for further analysis. Abbreviations for breeds are given in Table 1. Color codes for breeds are as in Figure 1.

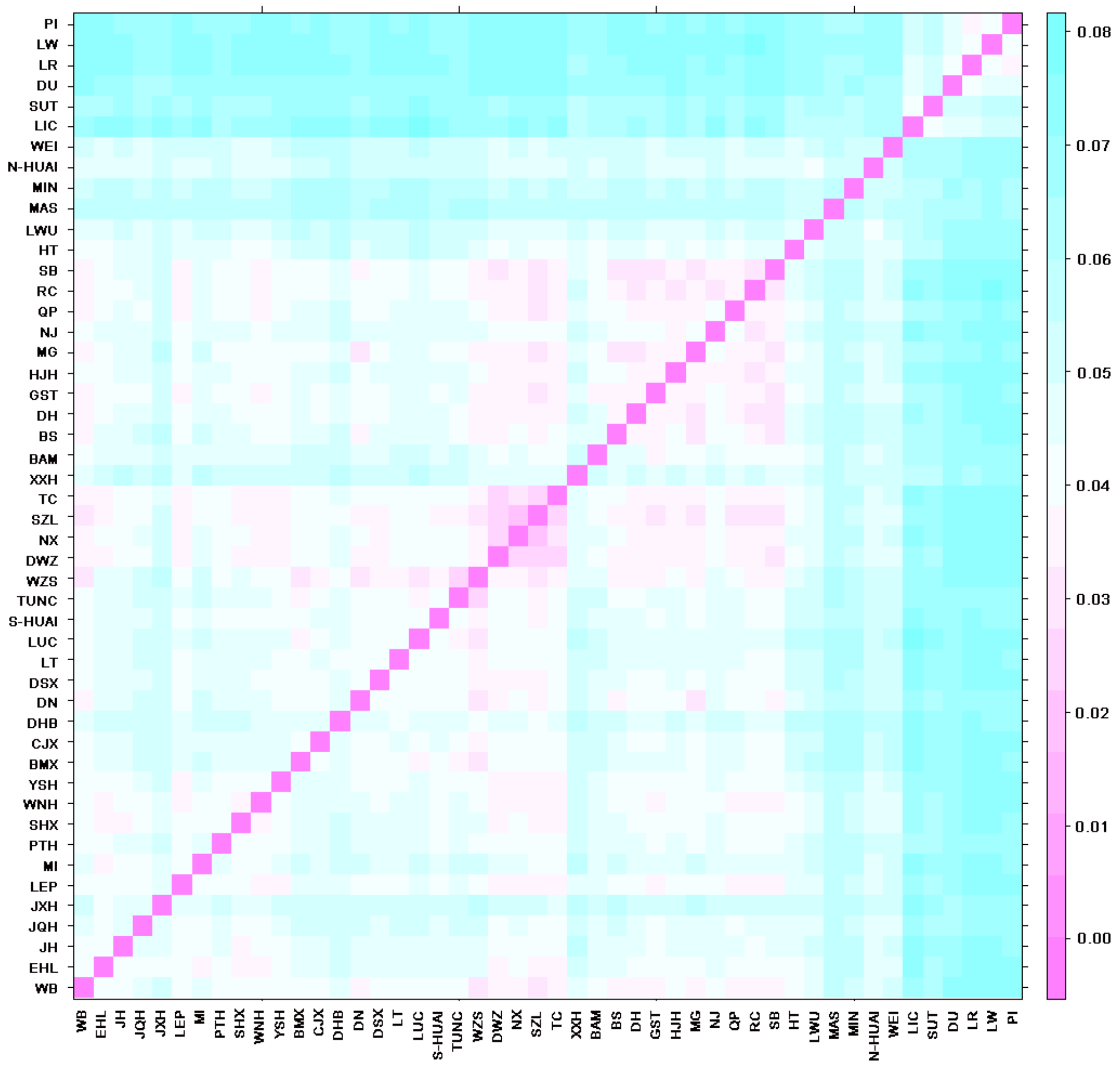

Figure S2 A heat map of pairwise Nei genetic distance amongst 47 Chinese and European domestic breeds and wild boars. Abbreviation for breeds and their ecotypes are given in Table 1.

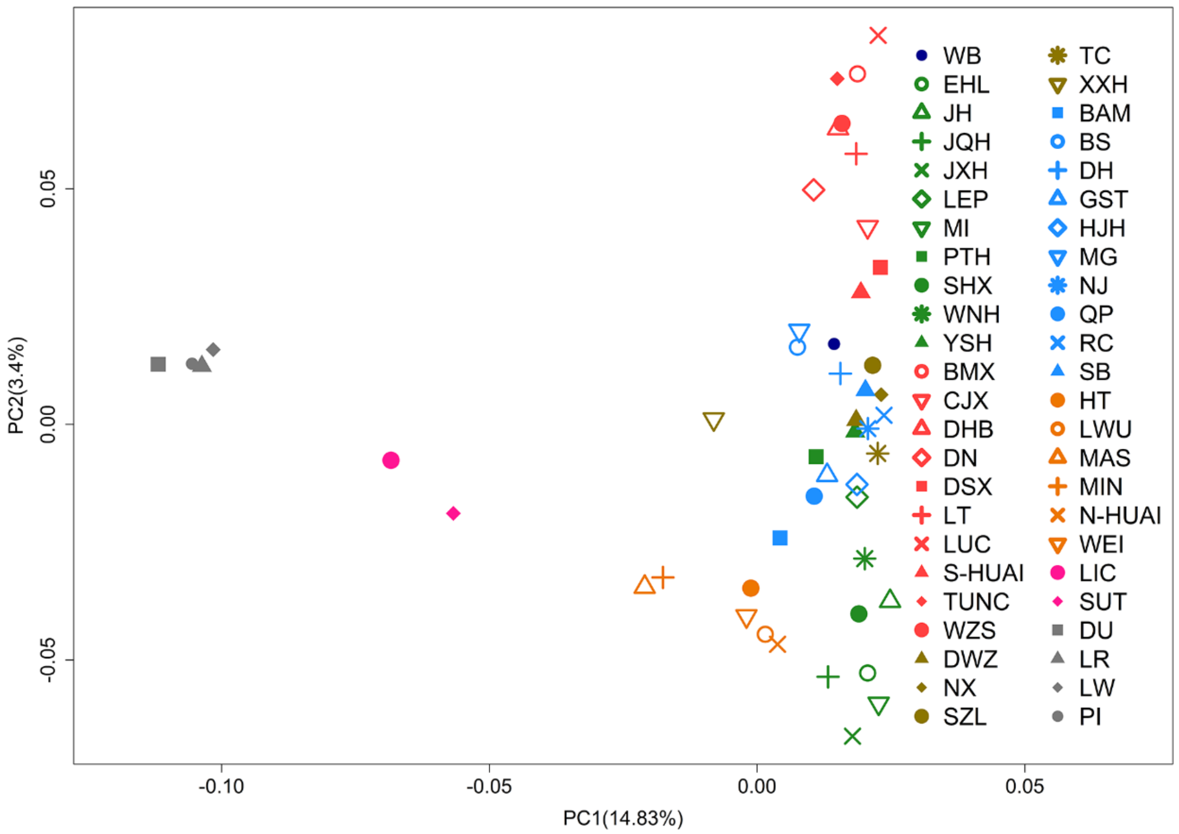

Figure S3 Principal component analysis of 48 Chinese and European pig breeds. The first (PC1) and second component (PC2) are shown. Each figured point represents the average eigenvector of one breed.

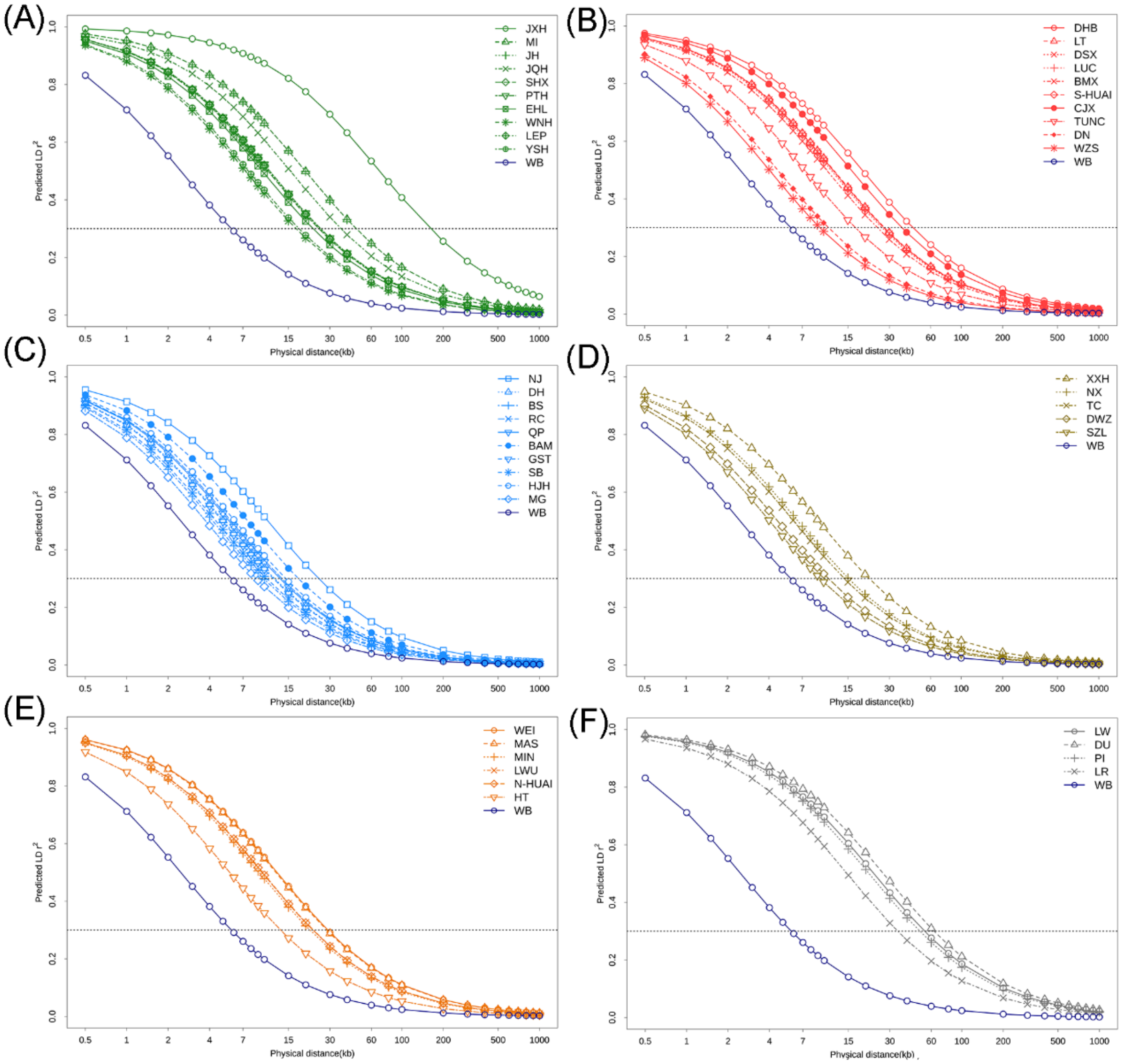

Figure S4 Linkage disequilibrium (LD) profiles in Chinese and European pigs. The profiles are shown for breeds from East China (A), South China (B), Central China (C), Southwest China (D), North China (E) and Europe (F). Wild boars are shown as an outgroup in each panel.

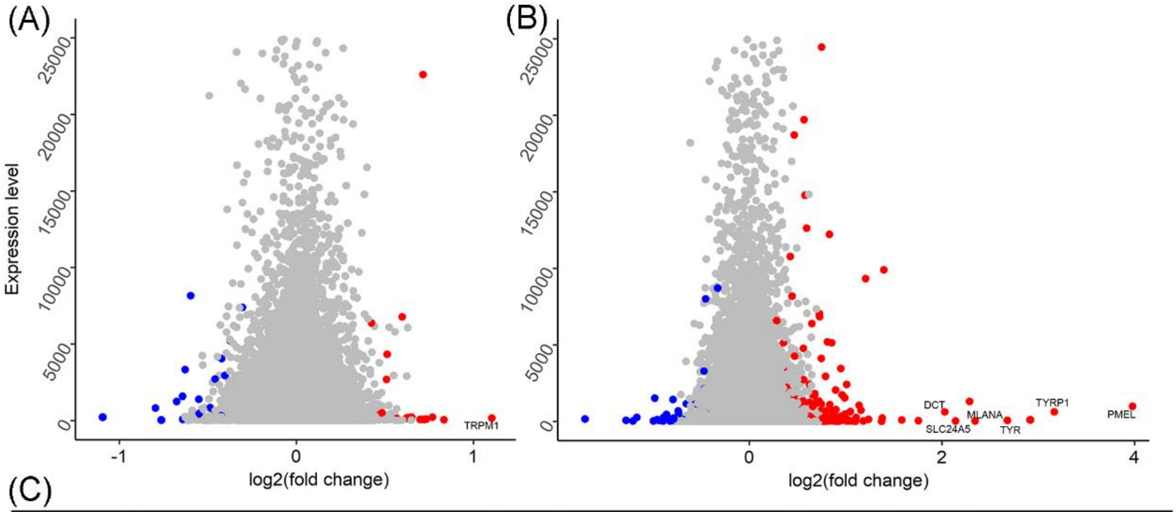

(C)

ID	GO Term	Ontology Source	P_{corr}	Associated Gene
GO:0048770	Pigment granule	GO_CellularComponent-GOA_23.02.2017_10h01	1.20E-07	*DCT*, *MLANA*, *PMEL*, *SLC24A5*, *TYR*, *TYRP1*
GO:0042470	Melanosome	GO_CellularComponent-GOA_23.02.2017_10h01	1.20E-07	*DCT*, *MLANA*, *PMEL*, *SLC24A5*, *TYR*, *TYRP1*
GO:0019748	Secondary metabolic process	GO_BiologicalProcess-GOA_23.02.2017_10h01	1.90E-07	*DCT*, *PMEL*, *SLC24A5*, *TYR*, *TYRP1*
GO:0042440	Pigment metabolic process	GO_BiologicalProcess-GOA_23.02.2017_10h01	4.30E-07	*DCT*, *PMEL*, *SLC24A5*, *TYR*, *TYRP1*
GO:0048066	Developmental pigmentation	GO_BiologicalProcess-GOA_23.02.2017_10h01	1.90E-06	*DCT*, *PMEL*, *TYR*, *TYRP1*
GO:0006582	Melanin metabolic process	GO_BiologicalProcess-GOA_23.02.2017_10h01	1.50E-09	*DCT*, *PMEL*, *SLC24A5*, *TYR*, *TYRP1*
GO:0018958	Phenol-containing compound metabolic process	GO_BiologicalProcess-GOA_23.02.2017_10h01	3.00E-06	*DCT*, *PMEL*, *SLC24A5*, *TYR*, *TYRP1*
GO:0044550	Secondary metabolite biosynthetic process	GO_BiologicalProcess-GOA_23.02.2017_10h01	2.20E-09	*DCT*, *PMEL*, *SLC24A5*, *TYR*, *TYRP1*
GO:0046148	Pigment biosynthetic process	GO_BiologicalProcess-GOA_23.02.2017_10h01	1.80E-07	*DCT*, *PMEL*, *SLC24A5*, *TYR*, *TYRP1*
GO:0042438	Melanin biosynthetic process	GO_BiologicalProcess-GOA_23.02.2017_10h01	1.20E-09	*DCT*, *PMEL*, *SLC24A5*, *TYR*, *TYRP1*
GO:0046189	Penol-containing compound biosynthetic process	GO_BiologicalProcess-GOA_23.02.2017_10h01	8.80E-08	*DCT*, *PMEL*, *SLC24A5*, *TYR*, *TYRP1*
GO:0005507	Copper ion binding	GO_MolecularFunction-GOA_23.02.2017_10h01	1.00E-04	*DCT*, *TYR*, *TYRP1*
GO:0033162	Melanosome membrane	GO_CellularComponent-GOA_23.02.2017_10h01	2.50E-06	*DCT*, *TYR*, *TYRP1*
GO:0045009	Chitosome	GO_CellularComponent-GOA_23.02.2017_10h01	2.50E-06	*DCT*, *TYR*, *TYRP1*
GO:0000350	Tyrosine metabolism	KEGG_01.03.2017	3.60E-05	*DCT*, *TYR*, *TYRP1*

Figure S5 Differentially expressed genes between black and white skin tissues of Bamaxiang and Jinhua pigs. (A) Bamaxiang pigs. (B) Jinhua pigs. (C) GO terms and KEGG pathways in which 34 differentially expressed genes in Jinhua pigs are enriched. Up- and down- regulated genes in the black skin are indicated by red and blue dots, respectively. Well-characterized pigmentation genes are highlighted in panels A and B.
